# Modeling MRD Changes in Myeloma to Understand Treatment Effects, Predict Outcomes, and Investigate Curative Potential

**DOI:** 10.1158/1078-0432.CCR-24-3475

**Published:** 2025-03-27

**Authors:** Walter M. Gregory, Thomas J. Prior, J. Blake Bartlett, Pieter Sonneveld, Meletios A. Dimopoulos, Philippe Moreau, Saad Usmani, Thierry Facon

**Affiliations:** 1 Division of Clinical Medicine, University of Sheffield, Sheffield, United Kingdom.; 2 Janssen Research & Development, Spring House, Pennsylvania.; 3 Janssen Research & Development, LLC, Raritan, New Jersey.; 4 Erasmus MC Cancer Institute, Rotterdam, the Netherlands.; 5 Department of Clinical Therapeutics, School of Medicine, National and Kapodistrian University of Athens, Alexandra General Hospital, Athens, Greece.; 6 Hematology Department, University Hospital Hôtel-Dieu, Nantes, France.; 7 Memorial Sloan Kettering Cancer Center, New York, New York.; 8 University of Lille, CHU Lille, Lille, France.

## Abstract

**Purpose:**

We designed mathematical models to describe and quantify the mechanisms and dynamics of minimal residual disease (MRD) in order to better understand these MRD dynamics; inform future treatment design, including when to stop or change treatment; and extrapolate from current progression-free survival (PFS) times to predict future PFS curves.

**Experimental Design:**

This study aims to model individual sequential MRD data from phase III clinical trials (MAIA, CASTOR, and POLLUX) using previously developed mathematical models, which will be modified as needed to accurately reflect the actual MRD data. These models will then be used to project PFS curves into the future.

**Results:**

Patients with low MRD values either showed rapid disease regrowth, or the MRD values remained low for a prolonged period. Treatment seemed to be most effective in terms of cell kill within the first 6 to 12 months. Regrowth rates were correlated with estimated initial residual disease, particularly in MRD-negative patients. Three-year model extrapolations of PFS were closely comparable with clinical trial data.

**Conclusions:**

This model could provide early prediction of PFS outcomes, which otherwise takes lengthy periods of time to observe in clinical trials. Patients showing rapid rebound from low MRD values may benefit from adding another treatment before reaching progressive disease. The MRD analyses and results presented, such as the results about efficacy occurring early in the first 6 to 12 months, may help guide the development and selection of optimal regimens. Longer follow-up periods and application to other trials and datasets are required to substantiate these findings.

**Translational Relevance**

Mathematical modeling of serial minimal residual disease (MRD) biomarker values over time in multiple myeloma allowed estimation of both the amount of resistant disease at treatment onset and the rate of subsequent regrowth. Surprisingly, these two factors were found to be correlated. Associated modeling results suggested that treatment effects occur early, probably within 6 to 12 months, and this provided insights into the timing of treatment effects in multiple myeloma, which inform trial design decisions about when to stop or change treatment. A new population-based mathematical model was developed from these findings, which incorporated the observed relationship between the quantity of initially resistant residual disease and its regrowth. The model accurately predicted long-term progression-free survival in multiple myeloma. Such use and modeling of continuous MRD values extend previous work looking at dichotomized MRD thresholds as they relate to PFS in multiple myeloma.

## Introduction

Therapeutic advances have significantly improved the survival of patients with multiple myeloma, with the median overall survival (OS) of standard-risk patients increasing from 2 to 4 years in the late 1990s and early 2000s (1–3) to 7 to 10 years (4, 5) currently. Given the extended time to collect mature data, there is a need for effective surrogate clinical endpoints, which may reduce costs and strain on resources while accelerating drug development and evidence-based guidance on therapy selection.

Although complete response (CR) correlates with improved patient outcomes (6), a large portion of patients now achieve CR. The assessment and monitoring of minimal residual disease (MRD), defined as the absence of tumor plasma cells within 1,000,000 bone marrow cells [limit of detection (LOD) <10^−6^], provide a more sensitive determination of disease burden (7). MRD-negative CR experienced significantly greater clinical benefits compared with MRD-positive CR, for which outcomes were similar to those achieving a lesser response (8, 9). Thus, MRD negativity is a strong predictor of disease outcomes (10).

In a comprehensive meta-analysis, the achievement of MRD negativity was strongly associated with improvements in both progression-free survival (PFS) and OS in a large cohort of transplant-eligible and transplant-ineligible patients with newly diagnosed multiple myeloma or relapsed or refractory multiple myeloma (11), making the achievement of durable and sustained MRD negativity a primary goal of multiple myeloma treatments. Furthermore, there is growing interest in and support for utilizing MRD as a clinically validated endpoint to predict PFS/OS (10, 12).

Although MRD status is an important marker for assessing treatment efficacy, PFS remains a primary endpoint in most clinical, and particularly registrational, trials because the goal of multiple myeloma treatment is to effectively extend survival and mitigate disease progression. Because a lengthy period is required to observe PFS, accurately predicting and projecting PFS curves would allow clinical trial results to be obtained more quickly.

Previous mathematical models have shown the potential to relate patient outcomes to underlying disease processes such as tumor regrowth rates and the number of subclinical resistant disease cells (13–16). Although these mathematical models exist both at a population level (13, 14, 16) and at an individual patient level (15), there is a need for alternative, updated models in which the assumptions of the model are informed, checked, and validated by individual patient MRD data in multiple myeloma, thereby improving the accuracy, relevance, and validity of the models.

In this study, we utilized individual sequential MRD data at an early stage to predict subsequent PFS curves at a later stage. In addition, we explored and characterized the dynamics of serial MRD values over time to better understand how they might inform the development and selection of optimal treatment regimens and ultimately influence multiple myeloma treatment practices.

## Materials and Methods

### Previous and new mathematical/statistical model

The previously published population-based mathematical model (13, 14, 16) related durations of long-term outcomes such as disease-free survival/CR to the amount and regrowth of underlying residual disease, assumed to be resistant at the start of treatment. The model was developed in and applied to a number of diseases, including leukemia, Hodgkin's disease, myeloma, and breast cancer. This previous population-based model assumed a lognormal distribution of residual/resistant disease, a lognormal distribution of tumor doubling times, that the residual/resistant disease regrows exponentially, and that the amount of residual/resistant disease at the start of treatment is independent of its subsequent regrowth. The justifications for these assumptions have been previously reported (13, 14, 16).

**Figure 1.**
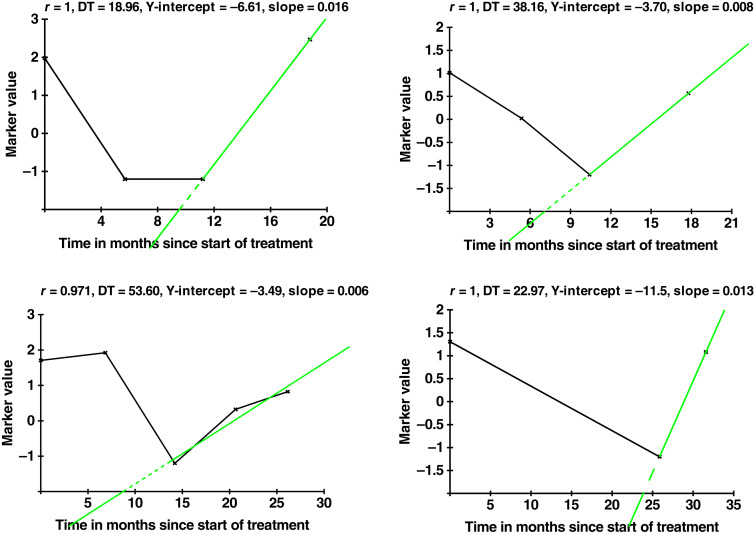
Example curves showing back-extrapolation of Y-intercepts and estimated growth rates from sequential MRD values. DT, doubling time; r, correlation coefficient. Note: Example results are shown for four individual patients. Green dashed lines represent back-extrapolation.

The mathematical model estimates a “Y-intercept” parameter for each patient whose disease regrows after treatment. This parameter is back-extrapolated from the later regrowth of the disease (Fig. 1) and is a potentially very low, hypothetical starting value for (resistant) disease growth. As in these patients, the disease is regrowing despite treatment, this is effectively the volume of resistant disease at the start of treatment that will subsequently regrow and cause progression. The lowest MRD value biologically achieved by that patient occurs slightly later as sensitive disease is eliminated by treatment, and this regrowing resistant disease becomes dominant (Supplementary Fig. S1). This lowest MRD value might not be observed in the trial because it may be below the LOD of the assay and/or because the intermittent timing of MRD assays for the patient did not allow for measuring the lowest value.

It might appear from these assumptions that we are hypothesizing that all the therapeutic effect happens very early on, with the consequence that continuing therapy unnecessarily exposes patients to treatment toxicity when the growth from remaining disease occurs despite continuing therapy. However, in the model in which treatment is assumed to kill sensitive cells but not resistant cells (15, 17, 18), eventually resulting in unchecked growth with all cells being resistant, there can be a long period in which the treatment is still effective by killing remaining sensitive cells. This model typically assumes that the proportion of cells resistant to therapy is initially extremely small—approximately one in 10⁶ or 10⁷. As a result, it can take a substantial amount of time before the resistant population expands to comprise the entire tumor, ultimately leading to a state of “unchecked growth” (19). It will appear that therapy is still working effectively, and it is. This effect can be seen in Supplementary Fig. S1, which looks at the effects of such long-term therapy on the sensitive and resistant cell populations for a range of different values for the (proportional) cell kill and shows the resulting durations of treatment efficacy.

**Figure 2.**
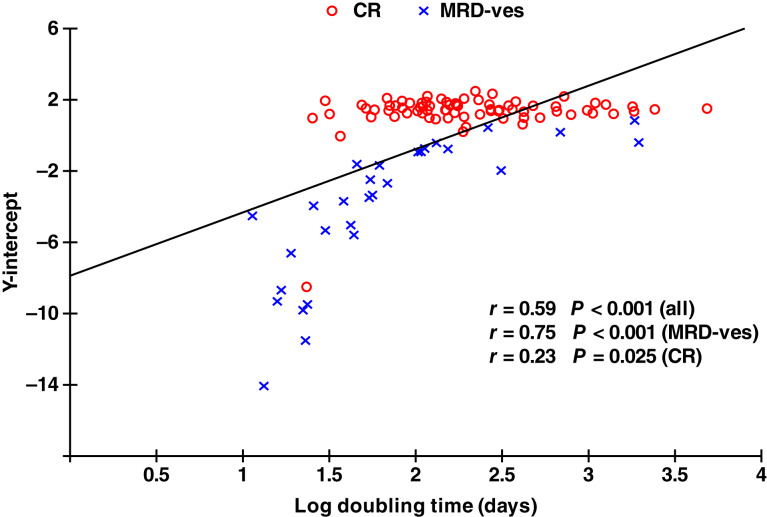
Y-intercept versus log doubling time (days). CR, complete response; MRD-ve, minimal residual disease-negative.

Examination of data from the MAIA (20), CASTOR (21), and POLLUX (22) trials demonstrated that there was a correlation between this “Y-intercept” and the subsequent regrowth of the disease, contrary to the original model assumptions (Fig. 2; Supplementary Figs. S2 and S3). Consequently, the original model, which employed the same exponential MRD growth rate for all patients, was generalized to incorporate this correlation, adding considerable complexity to the fitting process (see Supplementary Methods) but allowing the model to accommodate a wider class of patterns for serial values of a biomarker over time.

To fit the new model, data were simulated from a set of starting model parameters and smoothed to generate a simulated probability density function. A likelihood was then calculated from this probability density function. This process was then repeated using different model parameters, chosen based on the changing likelihood using a simplex algorithm (23), to definitively converge on the maximum likelihood estimate. A computer program for fitting and plotting both the original model and this revised model is available upon request.

### Studies included for analysis, modeling, and predictions

Initial analysis and modeling were based on data from the CASTOR and POLLUX trials in patients with relapsed/refractory multiple myeloma (21, 22). Primary analysis and modeling, however, were based on data acquired from the phase III MAIA trial (20) in patients with previously untreated, newly diagnosed multiple myeloma. Both POLLUX and MAIA compared the combination of daratumumab, lenalidomide, and dexamethasone (DRd) with the combination of lenalidomide and dexamethasone (Rd) alone. Both studies reported a substantial number of patients with sequential MRD measurements. Data acquired from the MAIA trial up to a clinical cutoff date of August 2018 were utilized for model development, and the PFS estimated curve was projected forward 3 years from this date. Data from subsequent updated analyses of the MAIA trial 3 years later were used to compare PFS curve predictions with actual MAIA results to validate the accuracy of the developed prediction model.

For all three trials, MRD was assessed using bone marrow aspirate samples by the clonoSEQ next-generation sequencing assay (Adaptive Biotechnologies, version 2.0). Samples were obtained at baseline, at the time of suspected complete or stringent CR, and, in all three trials for patients who had a CR or better, at 12, 18, 24, and 30 months after the first dose (MAIA trial); at 3 months after CR, 6 months after CR, and every 12 months after CR until the end of treatment (POLLUX trial); and at 6 and 12 months after the first dose and then every 12 months after CR until the end of treatment (CASTOR trial).

Due to the nature of the modeling/comparison analysis described in this article and the use of retrospective study data, no Institutional Review Board approval was needed, and this study was not listed on any clinical trial registry.

### Data availability

The data availability policy of Janssen Pharmaceutical Companies of Johnson & Johnson is available at https://www.janssen.com/clinical-trials/transparency. As noted on this site, requests for access to the study data can be submitted through the Yale University Open Data Access Project site at http://yoda.yale.edu.

## Results

### Individual patient MRD values

The numbers and timings of MRD measurements in the three trials MAIA, POLLUX, and CASTOR are given in detail in Supplementary Table S1. There was a very small number of values (about 4%) that were less than the 10^–6^ LOD but only marginally below this limit. MRD measurements were available for just beyond 3 years from the start of treatment.

For the MAIA trial (Supplementary Figs. S4–S6), the distributions of growth rates and Y-intercepts were approximately lognormal. Distributions were clearer for the distribution of doubling times compared with the distribution of Y-intercepts and were clearer in patients with CR than in MRD-negative patients. As shown in Fig. 2, there was a strong correlation between growth rates and Y-intercepts for MRD-negative patients. This correlation was not present in those with CR (see also Supplementary Figs. S7 and S8). The correlation coefficient in MRD-negative patients was 0.75 (*P* < 0.001), which was used later in the development of the population-based model. A similar correlation coefficient (0.74; *P* < 0.001) was observed in MRD-negative patients in the POLLUX data (Supplementary Fig. S2). A slight relationship (*r* = 0.42; *P* = 0.02) was observed in the CASTOR data (Supplementary Fig. S3). When looking at the data from all three trials together (Supplementary Fig. S9), the results seem similar for similar ranges of Y-intercepts. In fact, the differences between the results in the different trials seem to relate to the proportions of patients having different depths of response. In the MAIA and POLLUX trials, there are higher proportions with very low Y-intercepts, say <10^−3^, with no values below this level in the CASTOR data (25/137 vs. 0/28, *P* = 0.03, Fisher’s exact test). This may have been due to the poorer baseline prognosis of patients in the CASTOR trial limiting the number of MRD-negative patients, or to the fact that these patients have been previously treated, or to do with the use of immunomodulatory drugs in the MAIA and POLLUX trials compared with the use of a proteasome inhibitor in the CASTOR trial. As a proportion of patients in the POLLUX trial of previously treated patients seems to reach these very low levels, having had previous treatment seems unlikely to be the explanation for this higher proportion with low Y-intercepts, and this difference in the depth of response seems more likely to be a combination of the poorer baseline prognosis of patients and/or the use of a proteasome inhibitor in the CASTOR trial.

Across all three trials, most patients with two or more MRD measurements—enabling estimation of doubling times—had only two measurements. As a result, doubling time estimates in this group were more variable, with a standard deviation of 0.581 (110 patients) compared to 0.482 in patients with more than two measurements (>2 measurements, 77 patients; *F* = 1.45, *P* <0.01). This would explain some of the variability in the correlations between Y-intercepts and regrowth rates, and so the true correlation may well be even higher than the values shown here.

To ensure that patients who did not have two or more MRD values in an upward sequence (group A) did not have a different pattern of regrowth rates from the other patients (group B), additional analyses were conducted. These analyses examined the time from the last MRD measurement to progression in CR who did not reach the LOD. Patients were only included in this analysis if their last MRD values were in the range of the group A patients, allowing for a balanced comparison. There was no significant difference in the time from the last MRD measurement until progression between patients in groups A and B (*χ*^2^ = 0, *P* = 1, *n* = 36 and 24, respectively, log-rank test), indicating similar patterns of growth rates. A similar nonsignificant result was seen in the equivalent MRD-negative patients.

Summarizing, MRD-negative patients displayed a correlation with starting residual/resistant disease values that was either very limited or not present in MRD-positive CR, as well as displaying faster disease growth rates [median estimated log doubling times in the MAIA and POLLUX trials for MRD-negative and MRD-positive patients, respectively: 1.74 (55 days, *n* = 57) vs. 2.25 (178 days, *n* = 102), *z* = 4.7, *P* < 0.00001, Mann–Whitney test]. These observations suggest that there may be varied disease dynamics in patients with very low MRD volumes. Therefore, a more detailed analysis of MRD values in MRD-negative patients was performed, focusing on patients whose MRD values were below the LOD.

**Figure 3.**
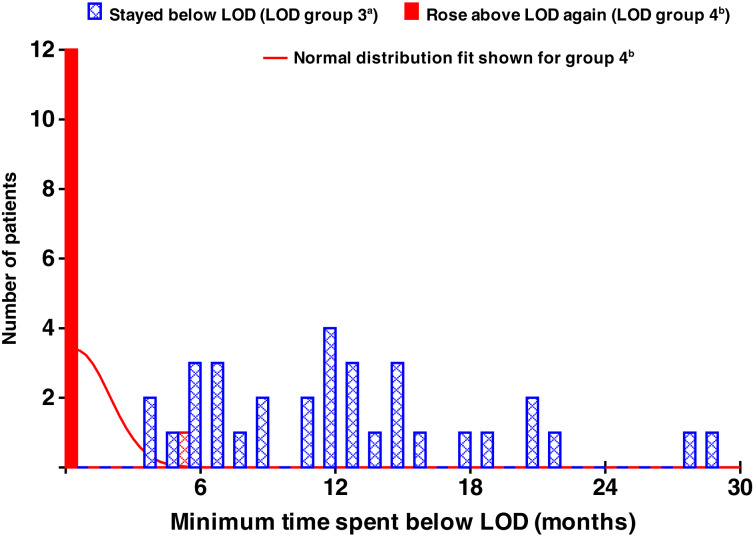
Minimum time spent below the LOD (months) by whether values increased above the LOD again or not. ^a^LOD group 3, values go below the LOD and stay below the LOD (≥2 values below the LOD). ^b^LOD group 4, values at or below the LOD and increase back above the LOD.

Four patterns (categories) of MRD change were observed in MRD-negative patients: (i) MRD values never reached the LOD; (ii) MRD values reached the LOD, but there were no further measurements; (iii) MRD values went below the LOD and remained there; and (iv) MRD values went below the LOD but then increased back above the LOD (Supplementary Figs. S10–S13, respectively). The number of patients in each observed category per treatment arm (DRd vs. Rd) is summarized in Supplementary Table S2. Only patients in categories 1 and 4 had an increasing sequence of MRD values that enabled the calculation of subsequent regrowth rates. The mean log doubling time in category 1 was 130 days, compared with 29 days in category 4 (*t* test, *t* = 3.50; *P* < 0.001). This suggests that patients who reach the LOD may present with a more rapidly growing disease compared with those who are MRD-negative but never reach the LOD range. Twelve out of 13 patients (92%) in category 4 had MRD values that increased back above the LOD immediately, and one patient had an MRD value that increased back above the LOD after a short interval of 5 months. In contrast, most category 3 patients (29/33; 88%) remained at or below the LOD for >6 months, with a mean (range) of 12 (1–29) months (Fig. 3); however, progression did occur in a small proportion of these patients (see below and Supplementary Fig. S14).

In the DRd arm of the MAIA study, of the 61 patients who reached LOD, 11 patients immediately returned from LOD (demonstrating the presence of bone marrow disease), and 23 remained at or below the LOD for >6 months (indicating the absence of disease). Of the remaining 27 patients, 21 reported final measurements at the LOD, and six remained at or below the LOD for <6 months; therefore, these final 27 patients can be categorized based on the 34 patients who have definitive results. Considering data from these 34 patients, 23 (68%) remained at or below the LOD, so it was assumed that 68% of these remaining 27 patients (18 patients) would have MRD values at or below the LOD for >6 months. Therefore, the estimated total proportion of patients with long-term MRD negativity below the LOD in the DRd arm is approximately 41 out of a total DRd population of 368 patients (11%). Among MRD-negative patients [median (range) follow-up, 2.4 (1.7–3.4) years], the estimated proportion with long-term MRD negativity below the LOD is 41/89, or about 46%. Similar calculations in the Rd control arm suggested rates of about 2% overall and about 22% for the MRD-negative patients.

To investigate the accuracy of these estimates, PFS curves were calculated for these four observed groups/categories using primary and updated data (an additional 3 years of follow-up), as data became available. The median (range) follow-up of the updated analyses was 5.5 (2.6–6.4) years. Of the 33 patients in category 3 from primary analysis data, 27 patients remained disease-free (Supplementary Fig. S14). With the inclusion of additional data from the extended follow-up, the total number of patients within category 3 increased to 47. In this updated patient category, only six patients displayed signs of progression, with a higher, more pronounced percentage of patients (80%) surviving without progression (Supplementary Fig. S15). With updated data, overall estimates for the proportion of patients with long-term MRD negativity below the LOD were 13% and 2% for DRd and Rd, respectively.

**Figure 4.**
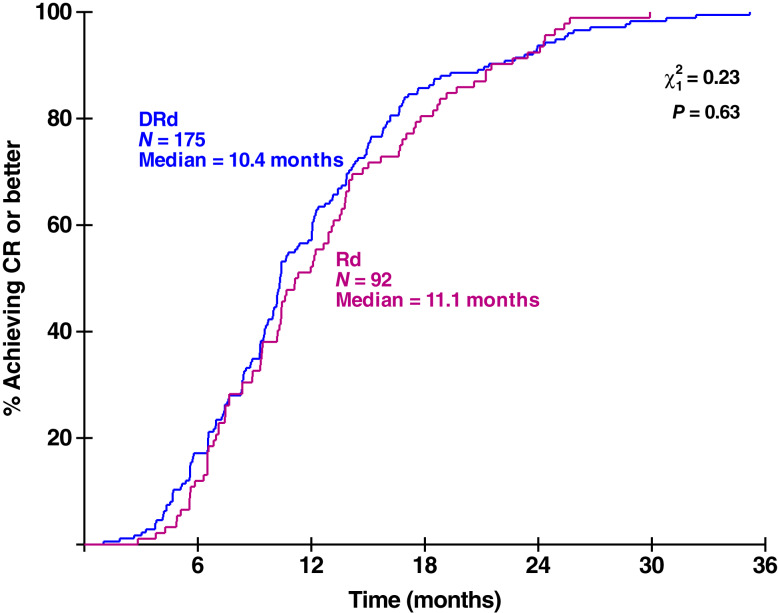
Time to achievement of CR or better by arm in patients achieving CR or better.

The rate of fall of MRD was calculated and plotted using available data from patients who had ≥2 MRD measurements in which the MRD value decreased following the start of treatment. Rates of fall were observed to be similar between treatment arms (DRd, *n* = 81; Rd, *n* = 21; *t* test, *t* = 0.42; *P* = 0.34). A total of 17 patients reported ≥3 downward measurements (15 reported three downward measurements and two reported four downward measurements). The observed fall was very clearly log-linear, with correlation coefficients averaging 0.997 (range, 0.988–1.0). These data suggest that MRD values decay exponentially during and following treatment, before increasing again or remaining at or below the LOD level. This lack of difference between the two treatment arms (DRd vs. Rd) was similar when plotting the time to achievement of CR or better (*χ*^2^ = 0.23; *P* = 0.63; log-rank test; Fig. 4), with comparable results also observed in the POLLUX trial (Supplementary Fig. S16). In the CASTOR trial, patients seemed to achieve CR slightly faster in the control arm, although caution should be used when making inferences because the sample size of patients achieving CR in this arm was small (Supplementary Fig. S17).

PFS curves were first modeled for each response subgroup before combining responses into one final, overall PFS prediction curve. Given the comprehensive and consistent MRD data in the CR group, this group was key, and parameters derived from the CR group were also used to inform the model fitting in the very good partial response and partial response groups. Although parameters were not as meaningful, they were also utilized for the no-response and unknown groups. Because there was no observed correlation in CR between the estimated Y-intercept and regrowth rates, it was plausible to apply the original model for patients with CR (Fig. 2; Supplementary Figs. S7 and S8). For the MRD-negative patients with a correlation between the Y-intercept and regrowth rate parameters, the new model (incorporating the correlation between these two factors) was used. As stated previously, the CR provided approximate estimates for the parameters in the other groups, namely, a mean doubling time of about 200 days (from CR) and SD of about 0.4 for both the mean residual disease parameter and the log mean doubling time parameter. Using these approximate parameter estimates, most of the model parameters were fixed (SD of residual disease, mean doubling time, SD of doubling time, and correlation for the MRD-negative group); however, this was sometimes varied as described below. The model fit was obtained within each response subgroup by maximizing the remaining parameters, primarily the residual disease parameter. This strategy makes intrinsic sense because the main difference between the response categories is the depth of response. The full set of parameter estimates is given in Supplementary Table S3.

### Fitting the MAIA PFS curves and projecting them forward in time

To appropriately fit the model to the unknown response group, substantially faster doubling times were required. Similarly, the very good partial response DRd fit required a longer doubling time of about 250 days and a wider spread of doubling times (represented by a large SD of the doubling time value) to produce a reasonable model fit. The fit in the MRD-negative patients used doubling time estimates and SD from the acquired individual data, along with values for the proportions of patients with long-term MRD negativity derived in the previous section of this article. The model fits for each response subgroup and by each treatment arm are displayed in Supplementary Figs. S18–S20.

**Figure 5.**
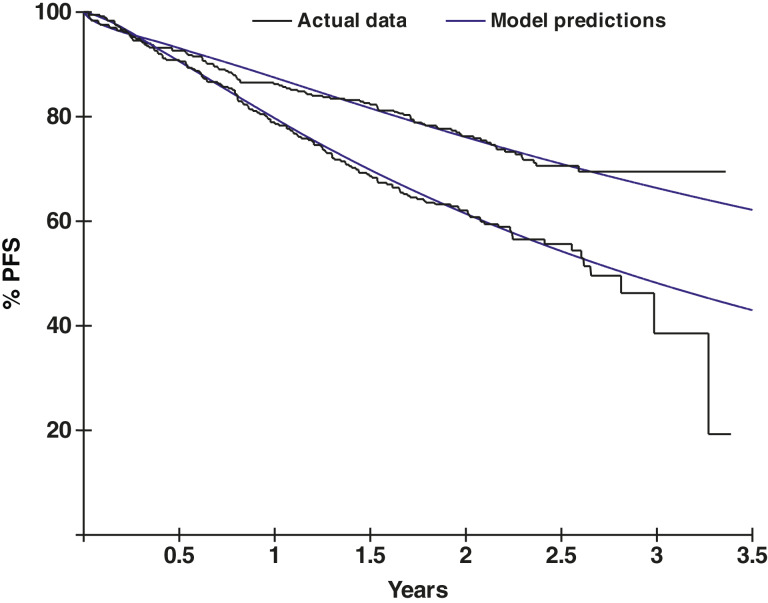
Overall PFS model fits to DRd and Rd arms, combining response subgroups. Kaplan–Meier curves are shown with censoring ticks removed.

Given the disparities between response groups—such as the differing numbers of patients lost to follow-up before approximately 20 months in the respective control arms and in the unknown and no-response groups—the resulting model curves could not be directly combined to generate an overall PFS predictive model. Thus, a Kaplan–Meier approach was used, applying model progression parameters on each day to everyone at risk on that day. The resultant overall model fit by treatment arm is shown in Fig. 5. This model predicts the PFS curve trajectory over time, thus allowing projection estimates to be made. Associated model probabilities can be used to estimate the chances of progression on any particular day, and simulation methods can therefore be used to calculate confidence intervals for those projections. Specifically, 3-year PFS predictions for each treatment arm in the MAIA study are displayed in Supplementary Fig. S21. The additional projected follow-up was applied to each individually censored patient at the time they were censored. Projections were not applied to patients censored <20 months because these patients were assumed to be lost to follow-up.

**Figure 6.**
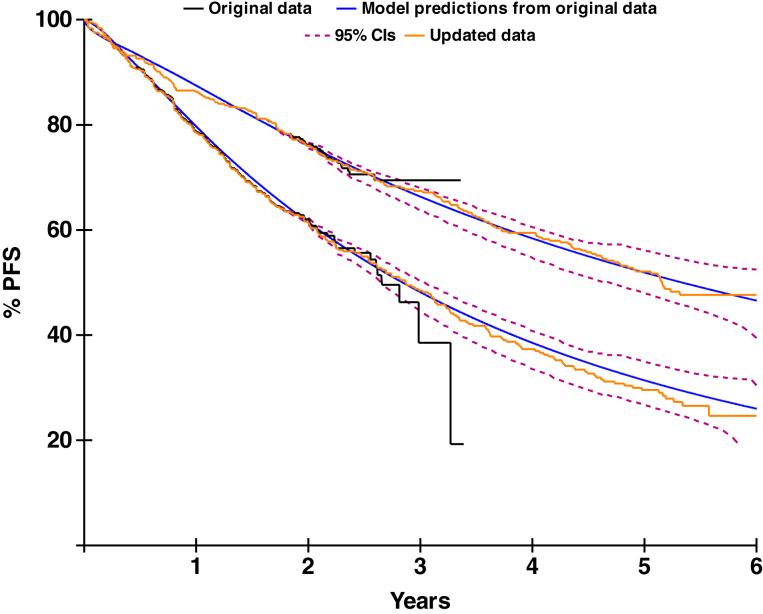
Model validation from the MAIA trial: PFS predictions from original data compared with actual results from updated data. CI, confidence interval. Kaplan–Meier curves are shown with censoring ticks removed.

After making these predictions using our developed PFS models, updated analyses from the MAIA trial were published (24), allowing for the direct comparison between actual clinical trial data and our model predictions with corresponding confidence intervals based on data from 3 years prior. As displayed in Fig. 6, there was little difference between the actual clinical results in the MAIA data and our model predictions, indicating very good model accuracy as well as the predictive power of the modeling approach. Data points were similar, and all predicted values were within the reported 95% confidence intervals.

## Discussion

With the therapeutic success of new drugs and multiple myeloma therapies over the past 20 years, patient survival times have notably increased. Thus, the focus is shifting to the possibility of a “cure,” how curative regimens can be designed, and the type of immunologic evidence necessary to optimize such designs. Results presented here demonstrate that evaluating very early patterns of MRD reduction and regrowth is crucial for understanding multiple myeloma disease progression. We found that the reduction to a very low MRD value for a short but sustained period may be sufficient to elicit beneficial disease outcomes in a substantial proportion of these patients. Given that these observed patterns in MRD occur so early (i.e., within 6–12 months), in-depth MRD analyses may provide crucial insights into individualized patient treatment responses. These early insights could identify patients who may benefit from adding another treatment to their treatment regimen before the disease regrows. Other recent in-depth analyses of MRD in multiple myeloma have also highlighted the relevance of early response, showing that the time to MRD resurgence is better in those reaching MRD negativity early, during induction (25), or before consolidation (26).

As shown in this study (Fig. 3), the number of diseased cells can increase from below the LOD at a rapid rate, within approximately 6 months. However, patients with sustained low levels of MRD beyond 6 months exhibit promising outcomes, indicating that a “cure” could potentially be achieved within this time frame of treatment. Modeling of the CASTOR and POLLUX trials and the MAIA trial, as reported here, suggests that daratumumab has a large cell-killing treatment effect in the first 6 to 12 months of treatment (27). Patient-level MRD data suggest that even some patients who become MRD-negative below the LOD may eventually exhibit regrowth of their disease. Treatment may have a higher cell-killing effect on more rapidly dividing cells, thus explaining disease behavior at these low disease levels (28). Therefore, the early individual disease growth rate may predetermine the efficacy of treatment, the ability to reach these very low MRD values, and the possibility of long-term MRD negativity or even “curing” multiple myeloma. This might be achievable by actively monitoring MRD and escalating or adding treatment at key early time points, including for patients with observable increases in MRD well before progressive disease occurs. Whether MRD negativity sustained beyond a certain period will equate to a cure in multiple myeloma is still unclear (29), although our data lend a degree of credence to this possibility. Note that the novelty in the LOD analysis is in showing that when the disease returns from being below the LOD, it returns very rapidly and very early; in these data, it returns within 6 months, and otherwise, it remains below the LOD for a substantial period of time.

As shown in the analysis of the initial downward slopes of MRD changes and the analysis of the time to achievement of CR by arm, response to treatment, when response occurs, typically happens within the first 6 to 12 months of treatment. Thus, in the case of MAIA, the addition of daratumumab increased the depth of response, as demonstrated here by the low MRD values in the DRd arm, the increase in the proportion of CR and MRD-negative patients in the daratumumab arm compared with the control arm, the possibility of long-term MRD negativity, and the substantially longer PFS for DRd versus Rd. These similar rates of fall by arm might seem counterintuitive, given the clear benefit of DRd over Rd. However, it seems likely that the fall in MRD levels is an exponential decay resulting from the treatment having already killed nearly all the viable malignant cells. This exponential decay would inevitably follow a similar pattern for any sufficiently effective treatment with a high cell kill. It would presumably continue until it encounters regrowing resistant disease, which would occur at different times for DRd compared to Rd because of the varying depths of response achieved in the two arms.

It might be argued that a limitation of this study is that it uses data from three clinical trials: two in relapsed/refractory multiple myeloma and one in newly diagnosed transplant-ineligible myeloma, and it does not include newly diagnosed transplant-eligible patients. However, the patterns of change in MRD are similar across all three trials, suggesting that the major differences between patients in these different groups are in the depth of response, that is, the proportions of patients achieving different depths of response are different in the three trials, but the patterns of regrowth and the relationship between the depth of response and the regrowth rate seem to be broadly similar. Furthermore, although the trials are all daratumumab-based, the control arms consist of non–daratumumab-based combination therapies, and the same patterns of change in MRD have been found in the control arms. Further work incorporating other trials, including newly diagnosed transplant-eligible patients, would help consolidate these findings and make the results even more generalizable.

Our analysis focuses on the dynamics of MRD changes over time, looking at both patterns in these changes, and the relationship to depth of response. The analyses do not take into account tumor biology, such as cytogenetic abnormalities and pretreatment prognostic factors, which may also be related to growth rates and depth of response. Such analyses would probably require a larger number and variety of trials, given that the patients for whom growth rate estimates are possible are limited, and cytogenetic subgroups, for example, can be small in size.

Optimizing the length of therapy use and the line of treatment in which a therapy is introduced is vital for developing effective treatment regimens. Understanding and dealing with resistance is crucial, and treatment strategies that are based on MRD dynamics, such as those used in the FLAIR trial (see below), have the potential to avoid or mitigate resistance and prevent overtreatment by optimizing the duration of treatment for an individual (30). Our current modeling analysis results present a window of opportunity to design studies that optimize the combinations and durations of novel therapies to attain maximum durable MRD negativity, thereby identifying a regimen that maximizes the potential curative patient fraction. Alternative strategies in which daratumumab is either halted at some point or treatment is switched/intensified, depending on the MRD dynamics, may be an interesting treatment strategy for future investigation. The optimum time to do this likely depends on the treatment regimen. In the PERSEUS trial (27), although not designed to address the question of optimal duration, approximately two-thirds of patients who achieved durable MRD negativity after 2 years of daratumumab plus lenalidomide maintenance, after DVRd induction and autologous SCT, were able to stop daratumumab. The impressive results of the FLAIR trial (31), in chronic lymphocytic leukemia, which were based on similar modeling principles, show the possibilities of such a modeling approach in a different disease. In FLAIR, treatment was continued for twice the time taken to achieve an MRD-negative remission.

In addition to our results providing insight into the dynamics of MRD status and its impact on disease progression, our developed model successfully predicted future PFS in the MAIA trial. PFS predictions based on MRD characteristics were comparable with the reported clinical trial data, with PFS estimates falling within the associated confidence intervals. This supports the notion that mathematical models, much like the one presented here, could be used to help predict long-term PFS outcomes at an earlier stage of a clinical trial. These predictions of patient PFS outcomes could thereby contribute to the production of valuable interim trial results, potentially shortening the duration of clinical trials and accelerating both research and drug development. Although MRD is already understood to be highly clinically meaningful and prognostic for long-term clinical outcomes (32), these additional approaches may extend its usefulness for disease progression estimation and prediction of patient responses.

In summary, the present MRD model supports the use of MRD dynamics and mechanisms to determine disease progression and to predict long-term clinical outcomes, including potential cure fraction. Our model highlights how changes in MRD often occur very early following treatment and that a reduction to very low and sustained MRD values for a relatively short period is prognostic of long-term beneficial outcomes. This insight may aid in the optimization of multiple myeloma regimen design and better inform the appropriate selection of treatment and prevent unnecessary overtreatment. Additionally, our MRD model enabled us to project PFS estimates into the future and accurately predict PFS outcomes. Thus, mathematical models like ours could be utilized to predict long-term patient outcomes, producing vital clinical trial data that may assist in the acceleration of drug development and help guide early treatment decisions for the benefit of the patient. The application of this model warrants confirmation in future, prospectively designed clinical trials.

## Supplementary Material

Supplementary Data 1 supplementary online material, tables, figures, mathematics.
