# Supplementary material for: Modeling MRD Changes in Myeloma to Understand Treatment Effects, Predict Outcomes, and Investigate Curative Potential
Source: Clin Cancer Res. 2025 Mar 27;31(11):2154–61. doi: 10.1158/1078-0432.CCR-24-3475 (PMC12130798; doi:10.1158/1078-0432.CCR-24-3475)

Supplementary online material

1. Legends for supplementary tables and figures

2. Mathematical derivation of the revised model

3. Supplementary tables and figures

## 1. Legends for supplementary tables and figures

Supplementary table 1. MRD measurements: numbers and timings

Supplementary table 2. LOD for MRD-negative patients by treatment arm

Supplementary table 3. Estimated parameters (using the likelihood method) for the different model fits.

Supplementary figure 1. Hypothetical plots of changes in tumour volume over time for treatments having different magnitudes of proportional cell-kill, showing different times at which the treatment effects cease.

Supplementary figure 2. Correlation between back-extrapolated Y-intercept and log doubling time in the POLLUX trial.

Supplementary figure 3. Correlation between back-extrapolated Y-intercept and log doubling time in the CASTOR trial.

Supplementary figure 4. Distributions of log doubling times in the MAIA trial by arm and response

Supplementary figure 5. Distributions of log doubling times in the MAIA trial for MRD negative patients

Supplementary figure 6. Distributions of Y-intercepts in the MAIA trial by response and arm

Supplementary figure 7. Correlation between back-extrapolated Y-intercept and log doubling time for CRs only in the MAIA trial

Supplementary figure 8. Correlation between back-extrapolated Y-intercept and log doubling time for CRs only in the MAIA trial excluding a single outlier

Supplementary figure 9. Correlation between back-extrapolated Y-intercept and log doubling in the MAIA, POLLUX, and CASTOR trials.

Supplementary figure 10. Examples of patients where the MRD never reaches the Limit of Detection range (LOD code: 1) in the MAIA trial.

Supplementary figure 11. Examples of patients where the MRD values enter the Limit of Detection range but have no more values (LOD code: 2) in the MAIA trial.

Supplementary figure 12. Examples of patients where the MRD values go below the limit of detection and stay there (LOD code: 3) in the MAIA trial.

Supplementary figure 13. Examples of patients where the MRD values go below the limit of detection and rise back again (LOD code: 4) in the MAIA trial.

Supplementary figure 14. PFS plotted by the four Limit of Detection categories for MRD negative patients in the MAIA trial.

Supplementary figure 15. PFS plotted with updated data by the four Limit of Detection categories for MRD negative patients in the MAIA trial.

Supplementary Figure 16. Time to achievement of CR or better by arm for those achieving CR or better in the POLLUX trial.

Supplementary Figure 17. Time to achievement of CR or better by arm for those achieving CR or better in the CASTOR trial.

Supplementary figure 18. Model fits to MRD negatives & CRs by arm in the MAIA trial.

Supplementary figure 19. Model fits to VGPRs & PRs by arm in the MAIA trial.

Supplementary figure 20. Model fits to NRs & Unknown responses by arm in the MAIA trial.

Supplementary figure 21. Overall PFS (95% CI) model fits in both arms with predictions for 3 years beyond currently available data.

## 2. Mathematical derivation of the revised model.

The original model assumes exponential growth of an initial resistant tumour volume, *v* and is described in detail in Gregory et al 2016[1]. The modification is to allow a correlation between the growth rate and the resistant tumour volume. Let the random variables *V* and *G* be normally distributed and represent the log of the resistant tumour burden, with mean $\mu_v$ and SD $\sigma_v$, and the log of the tumour doubling time, with mean $\mu_g$ and SD $\sigma_g$, Thus

$$V \sim N(\mu_v,\sigma_v) \text{ and } G \sim N(\mu_g,\sigma_g). \qquad (1)$$

A combined simulation/likelihood approach was used to incorporate the correlation. To do this it was assumed, in the simulation model, that the mean log growth rate was linearly related to the mean resistant disease as follows:

$$\mu_{gs} = k + s\mu_{vs} \qquad (2)$$

$$\sigma_{gs} = f\sigma_g \qquad (3)$$

where:

$\mu_{gs}$ is the mean (log) growth rate for the correlation model to be used in the simulation

$\mu_{vs}$ is the mean resistant disease for the simulation

$\sigma_g$ is the SD of the log of growth rates as in the baseline model (1) above

$\sigma_{gs}$ is the SD of the (log) growth rate for the correlation model to be used in the simulation

and

f is a multiplying factor on the SD, $\sigma_g$, of the log of the growth rates to generate an appropriate $\sigma_{gs}$ for this simulation sample such that the total variance of the log of the growth rates, incorporating the variance accounted for by the correlation with the residual disease shown in equation (2) and the variance from $\sigma^2_{gs}$ shown in equation (3) is equal to $\sigma^2_g$ (see next paragraph).

To derive appropriate values for s, k & f requires the log mean doubling time, derived from this linear relationship with the residual disease, to have the same mean and the same standard deviation as in the original baseline model without the linear relationship. So it is necessary to choose values for s, k, f that yield identical values for these two means ($\mu_{gs}$, $\mu_g$) and, in addition, it is necessary for the SD of the log of the growth rates that comes out of the simulation (that has the mean as a correlate of the residual disease) to match the original SD. To achieve this end note that part of the variance in the log of the growth rates is being taken out/accounted for in the simulation run by forcing the mean log growth rate to be related to the mean residual disease (to a degree determined by the desired correlation coefficient - if the correlation coefficient was very high, like .95, nearly all of the variance would have been taken out, f would be small, for instance .1, and the log of the growth rate would be almost entirely determined by the residual disease; if the correlation coefficient was very low, like .25, very little of the variance would have been accounted for, and f would be close to 1). Therefore, it is necessary to alter the SD of the log of the growth rates in the simulation to account for this and produce an overall SD that matches the baseline SD, $\sigma_g$, from the original model - as in equations (1). The multiplying factor, f, enables this end to be achieved by modifying the SD of growth rates, $\sigma_{gs}$. Note that variances are additive in the situation depicted by equations (2) and (3). Further note that, if $D^2$ represents the variance of a random variable X, then:

$$D^2(aX+b) = a^2D^2(X) \qquad (4)$$

So the variance resulting from the $f\sigma_g$ component is equal to $f^2\sigma_g^2$, which, as a proportion of the variance related to $\sigma_g$, is therefore simply $f^2$. The component of the variance which is explained by the correlation, r, is $r^2$. However, the component of the variance in this situation is that which is not explained by the correlation, i.e. $(1-r^2)$, so

$$f^2 = 1 - r^2 \qquad (5)$$

and therefore

$$f = \sqrt{(1 - r^2)} \qquad (6)$$

Turning to the component of the variance which *is* explained by the correlation, denoting the variance from the $\mu_{vs}$ part of the $s\mu_{vs}$ component as $\sigma_r$, and the accompanying mean as $\mu_r$, then, applying equation (4) again, the variance from the full $k + s\mu_{vs}$ component = $s^2\sigma_r^2$, and this explains all the variance due to the correlation. Therefore, as a proportion of the $\sigma_g$ variance, this is equal to $r^2$, i.e.

$$s^2\sigma_r^2/\sigma_g^2 = r^2 \qquad (7)$$

and therefore

$$s = r\sigma_g/\sigma_r \qquad (8)$$

Remember that this $\mu_{vs}$ component is a potentially doubly truncated normal distribution, so deriving $\sigma_r$ is not straightforward, but can be done by numerical methods. Having derived s, k can be derived by simply noting that k effectively just repositions the mean, to restore it to being equal to $\mu_g$ when it has been shifted by $s\mu_r$, so

$$k = \mu_g - s\mu_r \quad (9)$$

Again, the derivation of $\mu_r$ is not straightforward and has to be achieved by numerical methods.

Having arrived at values for k, s, & f data can be simulated with the required correlation, and with the correct means, but, at this point, not fitting the PFS data. However, the simulated data consists of events at particular times in days and can therefore be used to generate a simulated probability density function (pdf). This can then be smoothed with a running mean smoother. The likelihood for the dataset under consideration can then be calculated for this set of parameter values. A simplex algorithm[6] was then used to converge on the maximum likelihood estimates. To achieve the required accuracy to enable the simplex algorithm to converge adequately on the maximum likelihood estimates it was necessary to simulate large datasets, of the order of $10^9$ times, and so the fitting process is slow. It takes of the order of 5 hours on a PC with an i7 chip running Windows 11.

A graph with a series of example curves showing the difference the correlation makes to the shape of the PFS curves is given below for information. It can be seen that larger values for the correlation coefficient cause more of an initial delay before the curves start falling, and that the fall occurs more rapidly, and flattens out more quickly to the final plateau.

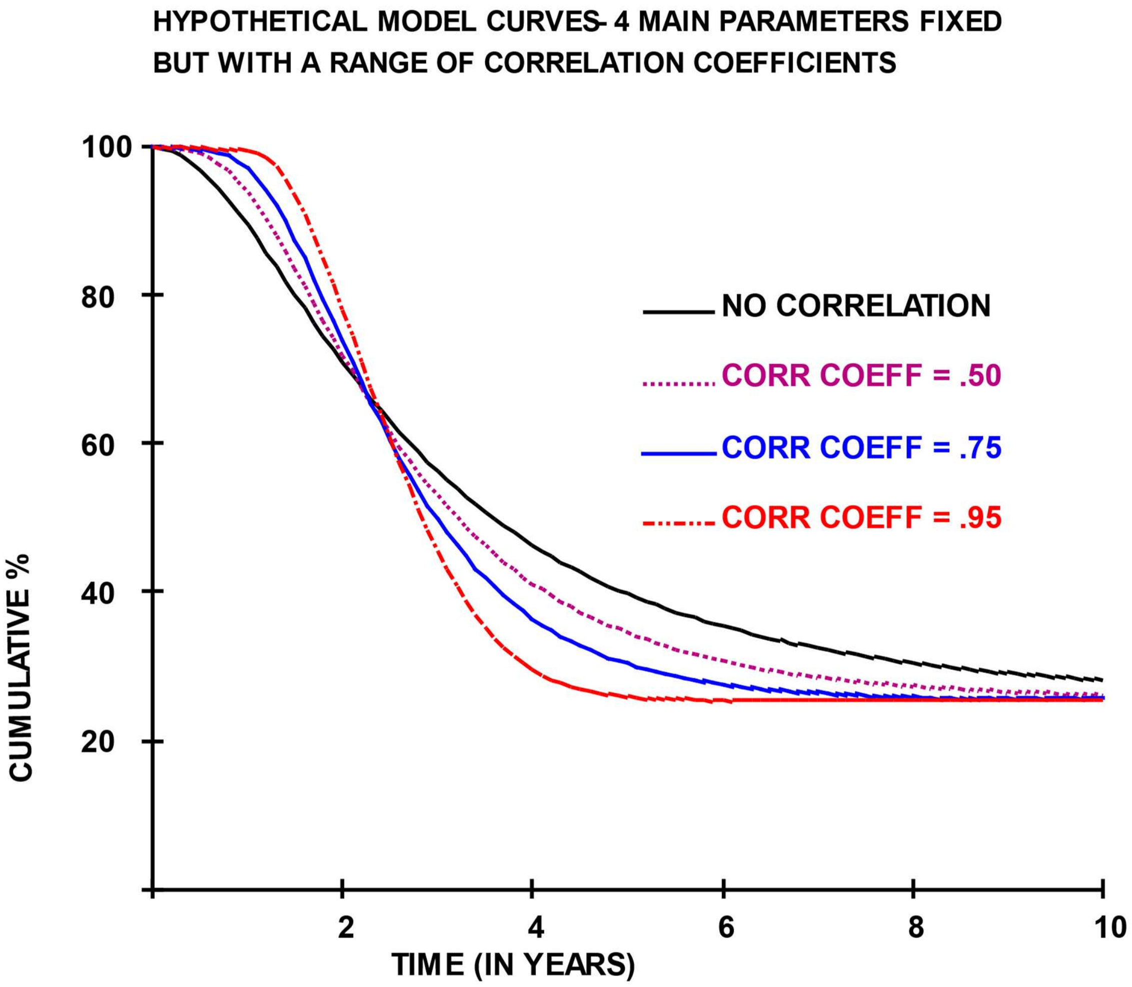

3. Supplementary tables and figures

**Supplementary Table 1. MRD measurements: numbers and timings**

| | MAIA | | POLLUX | | CASTOR | |
|---|---|---|---|---|---|---|
| **Total number of patients** | **238** | | **175** | | **84** | |
| **Total number of measurements** | **847** | | **659** | | **261** | |
| **Mean number of measurements per patient (range)** | **3.6 (1-7)** | | **3.8 (1-7)** | | **3.1 (2-6)** | |
| **Number & % of measurements <$10^{-6}$** | **29 (3.4%)** | | **32 (4.9%)** | | **7 (2.7%)** | |
| **Number of measurements < limit of assay** | **149** | | **179** | | **46** | |
| **Numbers of measurements within 6-month intervals from start of treatment** | **Month** | **Number** | **Month** | **Number** | **Month** | **Number** |
| | **6** | **41** | **6** | **58** | **6** | **54** |
| | **12** | **120** | **12** | **169** | **12** | **61** |
| | **18** | **178** | **18** | **121** | **18** | **24** |
| | **24** | **139** | **24** | **31** | **24** | **21** |
| | **30** | **103** | **30** | **49** | **30** | **10** |
| | **36** | **26** | **36** | **57** | **36** | **7** |
| | **42** | **2** | **42** | **2** | **42** | **0** |

**Supplementary Table 2. LOD for MRD-negative patients by treatment arm**

| LOD coding | DRd | Rd | Both |
|---|---|---|---|
| 1 | 28 (31%) | 17 (63%) | 45 |
| 2 | 21 (24%) | 4 (15%) | 25 |
| 3 | 29 (33%) | 4 (15%) | 33 |
| 4 | 11 (12%) | 2 (7%) | 13 |
| All | 89 | 27 | 116 |

LOD, limit of detection; MRD, minimal residual disease; DRd, daratumumab plus lenalidomide and dexamethasone; Rd, lenalidomide and dexamethasone

Note: Coding is as follows:

Code 1 patients are MRD negative (ie, $<10 \times 10^{-6}$) but never enter the LOD range.

Code 2 values enter the LOD range but no more values follow (ie, only a single LOD value).

Code 3 values go below the LOD and stay below the LOD (≥2 values below the LOD).

Code 4 values go at or below the LOD and rapidly rise back above the LOD.

**Supplementary Table 3. Estimated parameters (using the likelihood method) for the different model fits**

| Arm | Response subgroup | Mean residual disease | SD of RD | Mean doubling time | SD of doubling time |
|---|---|---|---|---|---|
| DRd | MRD-ve | 0.17 | 1.7 | 98 | 0.48 |
| Rd | MRD-ve | 1.3 | 1.7 | 103 | 0.48 |
| DRd | CRs | 5.0 | 0.4 | 200 | 0.4 |
| Rd | CRs | 6.1 | 0.4 | 200 | 0.4 |
| DRd | VGPRs | 7.0 | 0.4 | 250 | 0.5 |
| Rd | VGPRs | 7.2 | 0.4 | 200 | 0.4 |
| DRd | PRs | 8.0 | 0.4 | 200 | 0.4 |
| Rd | PRs | 7.8 | 0.4 | 200 | 0.4 |
| DRd | NRs | 9.0 | 0.4 | 200 | 0.4 |
| Rd | NRs | 8.7 | 0.07 | 200 | 0.4 |
| DRd | Unknown | 9.5 | 0.4 | 50 | 0.4 |
| Rd | Unknown | 8.8 | 0.4 | 50 | 0.4 |

DRd, daratumumab plus lenalidomide and dexamethasone; Rd, lenalidomide and dexamethasone; MRD-ve, minimal residual disease negative; CR, complete response; VGPR, very good partial response; PR, partial response; NR, no response; RD, residual disease; SD, standard deviation.

Supplementary figure 1. Hypothetical plots of changes in tumour volume over time for treatments having different magnitudes of proportional cell-kill, showing different times at which the treatment effects cease.

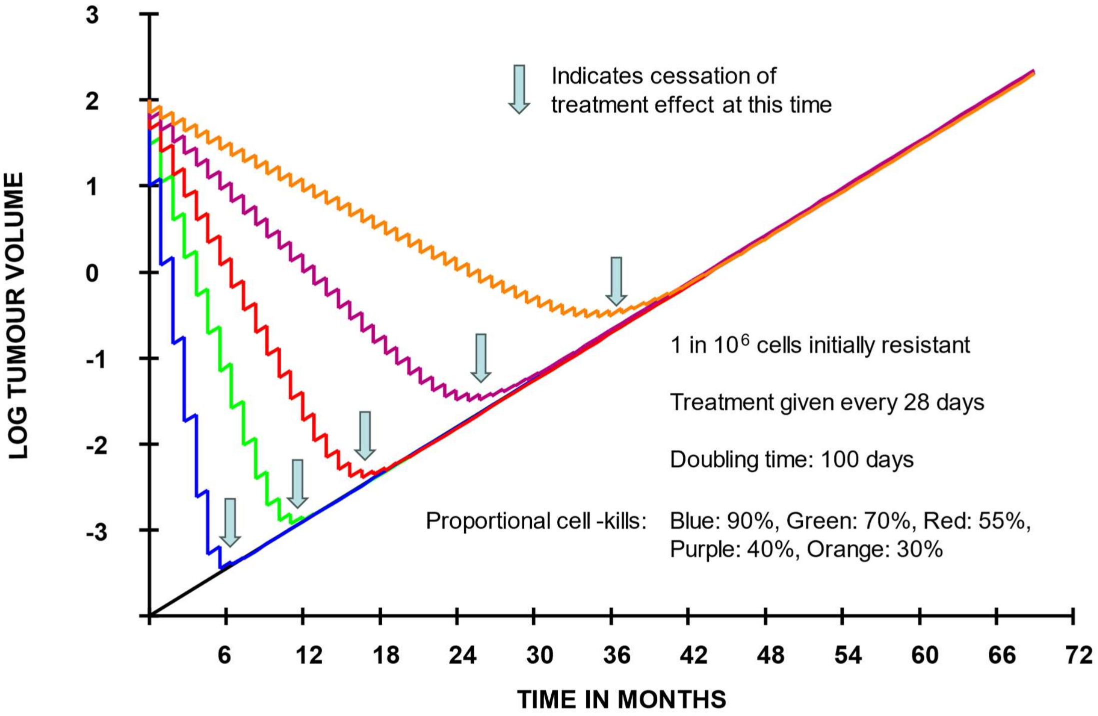

Supplementary figure 2. Correlation between back-extrapolated Y-intercept and log doubling time in the POLLUX trial.

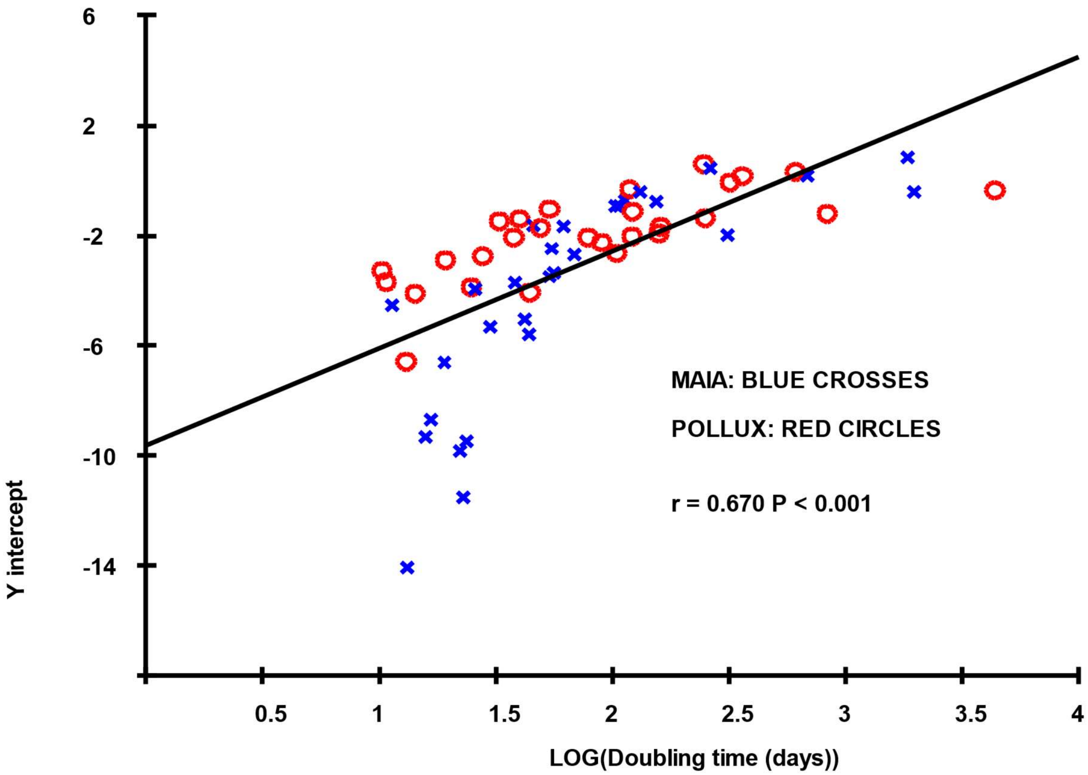

Supplementary figure 3. Correlation between back-extrapolated Y-intercept and log doubling time in the CASTOR trial.

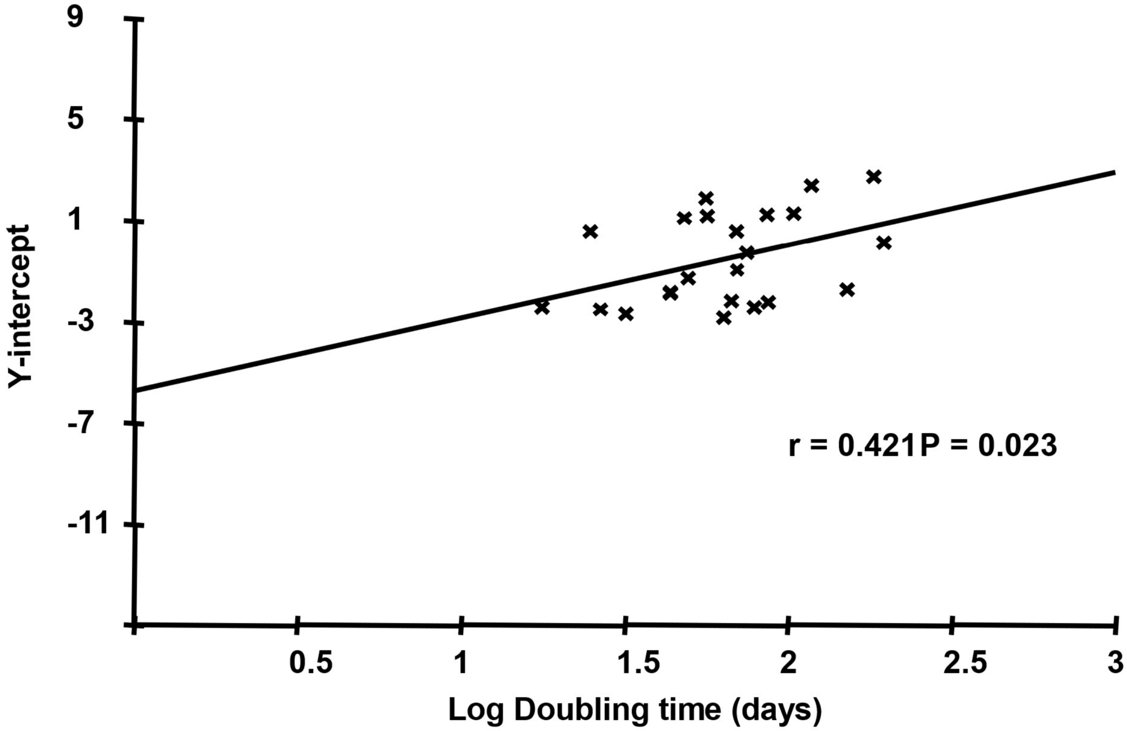

Supplementary figure 4. Distributions of log doubling times in the MAIA trial by arm and response

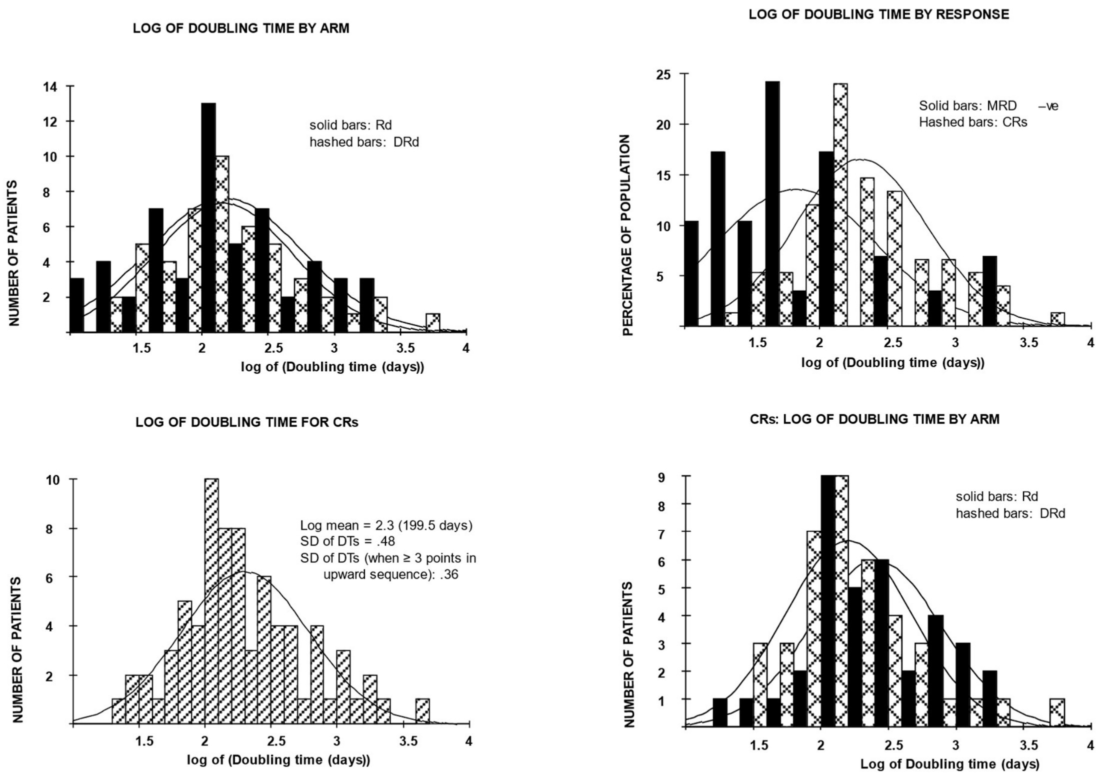

Supplementary figure 5. Distributions of log doubling times in the MAIA trial for MRD negative patients

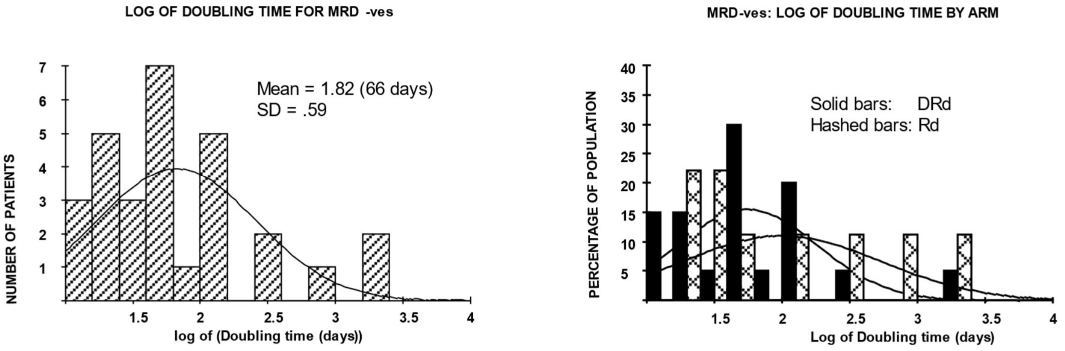

Supplementary figure 6. Distributions of Y-intercepts in the MAIA trial by response and arm.

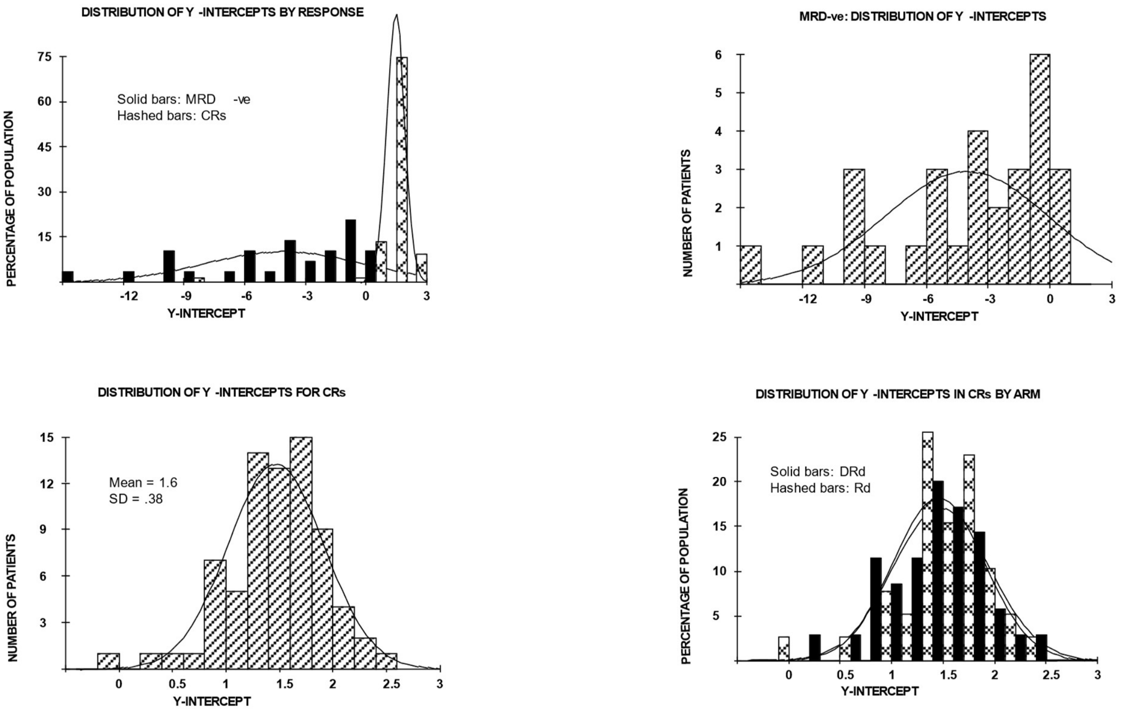

Supplementary figure 7. Correlation between back-extrapolated Y-intercept and log doubling time for CRs only in the MAIA trial

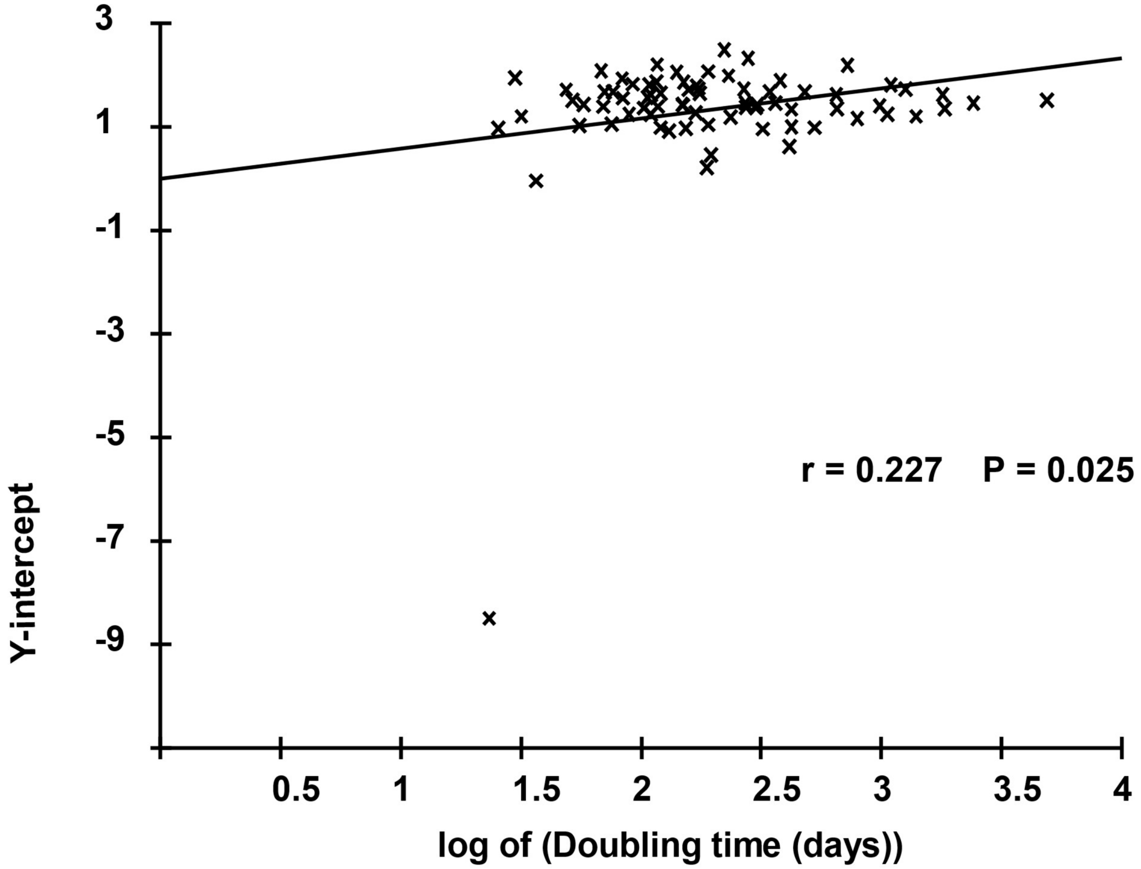

Supplementary figure 8. Correlation between back-extrapolated Y-intercept and log doubling time for CRs only in the MAIA trial excluding a single outlier

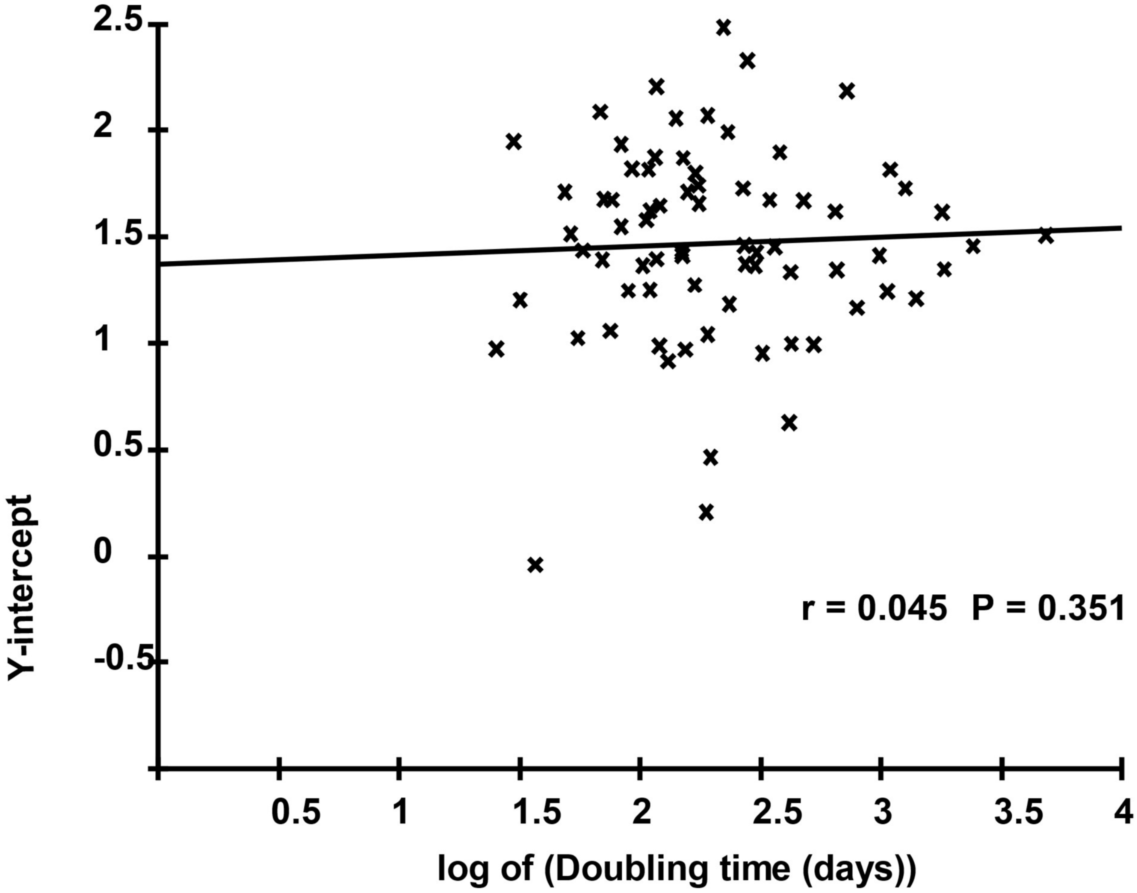

Supplementary figure 9. Correlation between back-extrapolated Y-intercept and log doubling in the MAIA, POLLUX, and CASTOR trials.

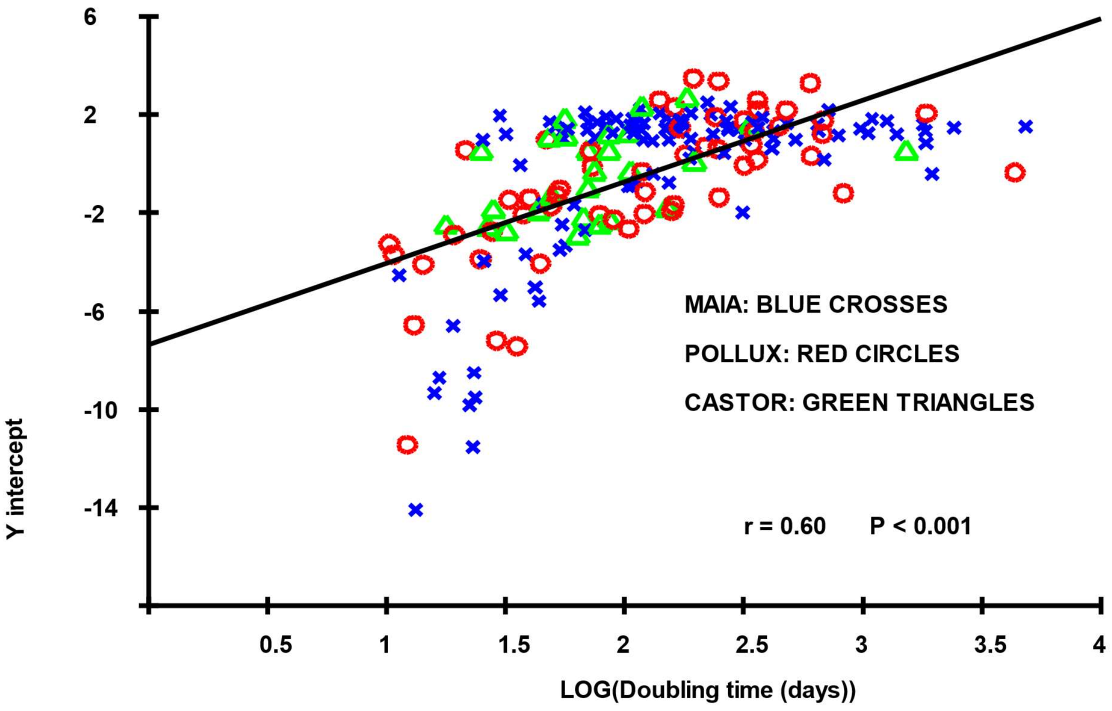

Supplementary figure 10. Examples of patients where the MRD never reaches the Limit of Detection range (LOD code: 1) in the MAIA trial.

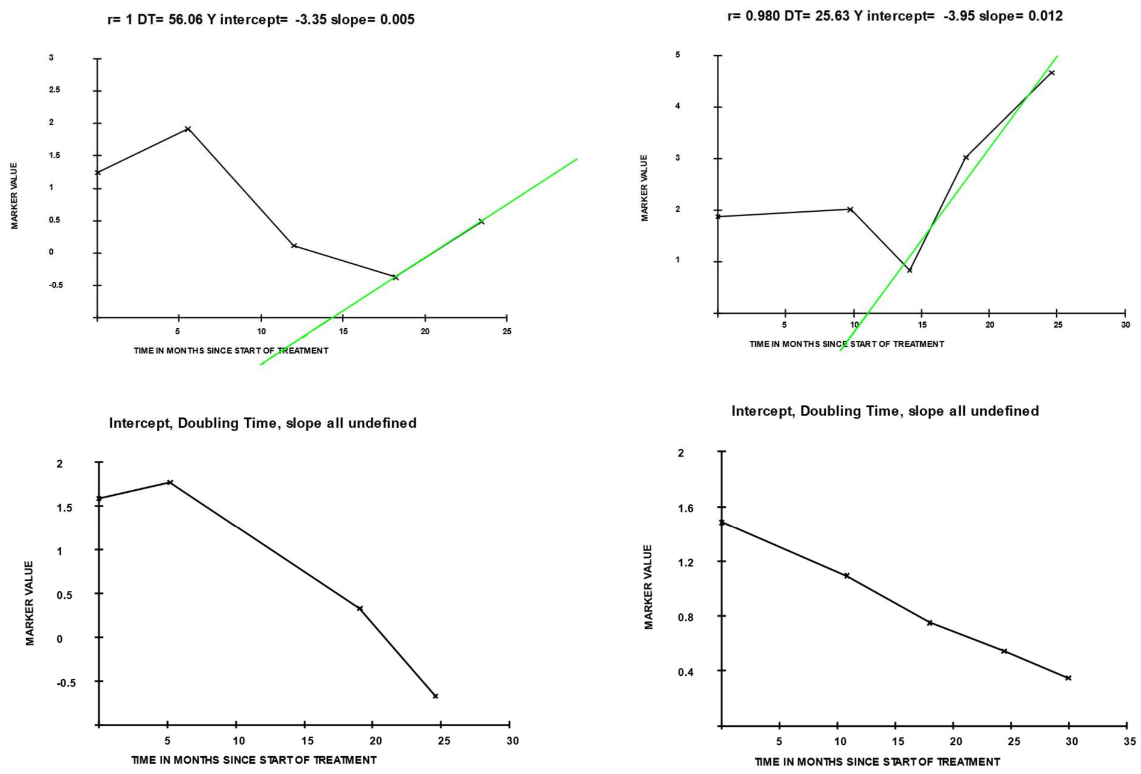

Supplementary figure 11. Examples of patients where the MRD values enter the Limit of Detection range but have no more values (LOD code: 2) in the MAIA trial.

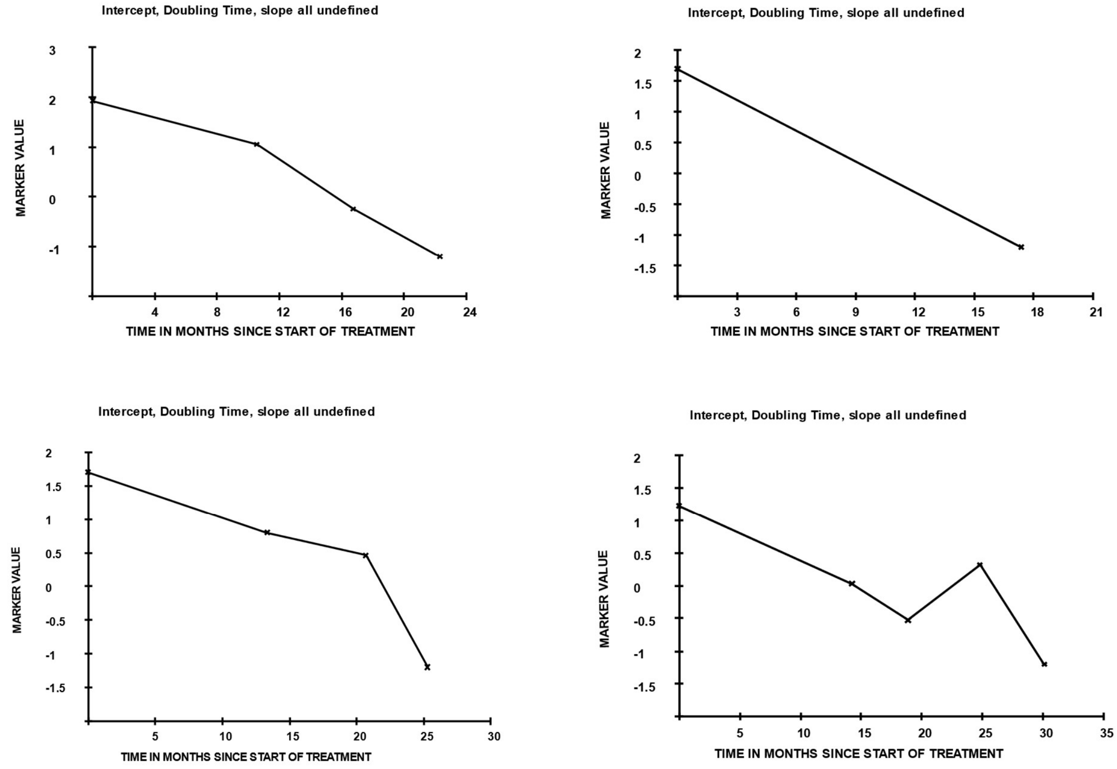

Supplementary figure 12. Examples of patients where the MRD values go below the limit of detection and stay there (LOD code: 3) in the MAIA trial.

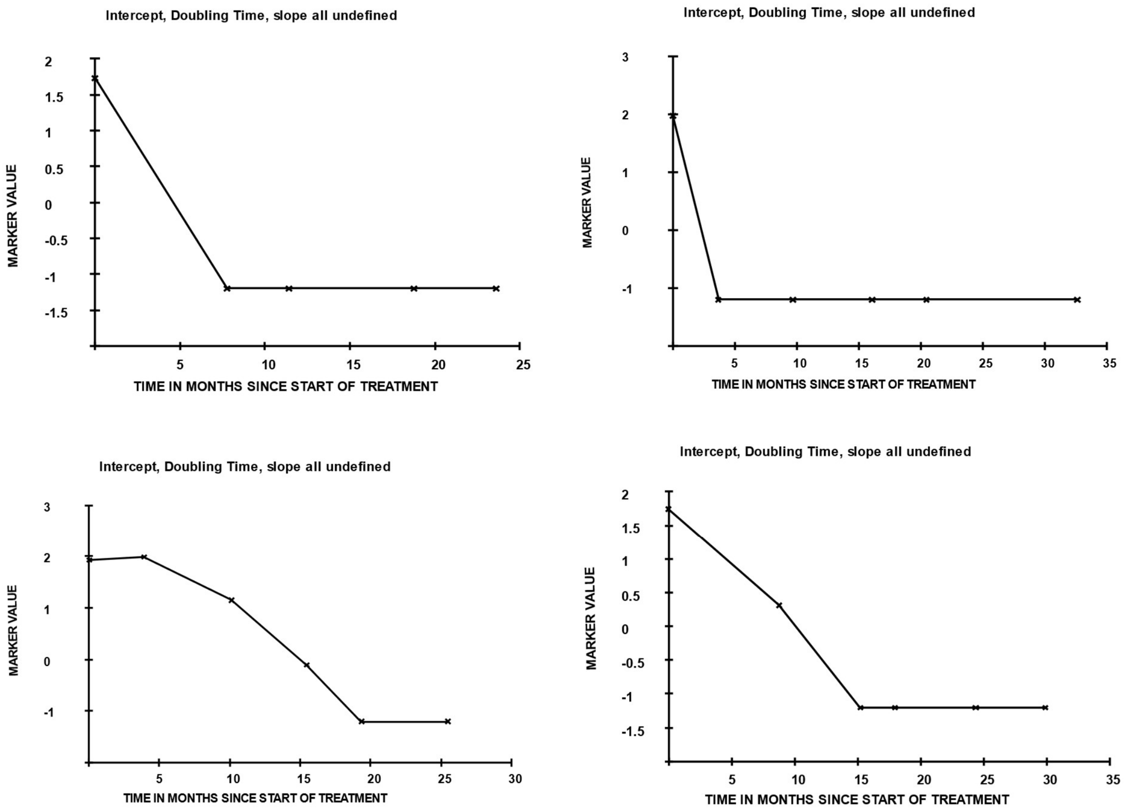

Supplementary figure 13. Examples of patients where the MRD values go below the limit of detection and rise back again (LOD code: 4) in the MAIA trial.

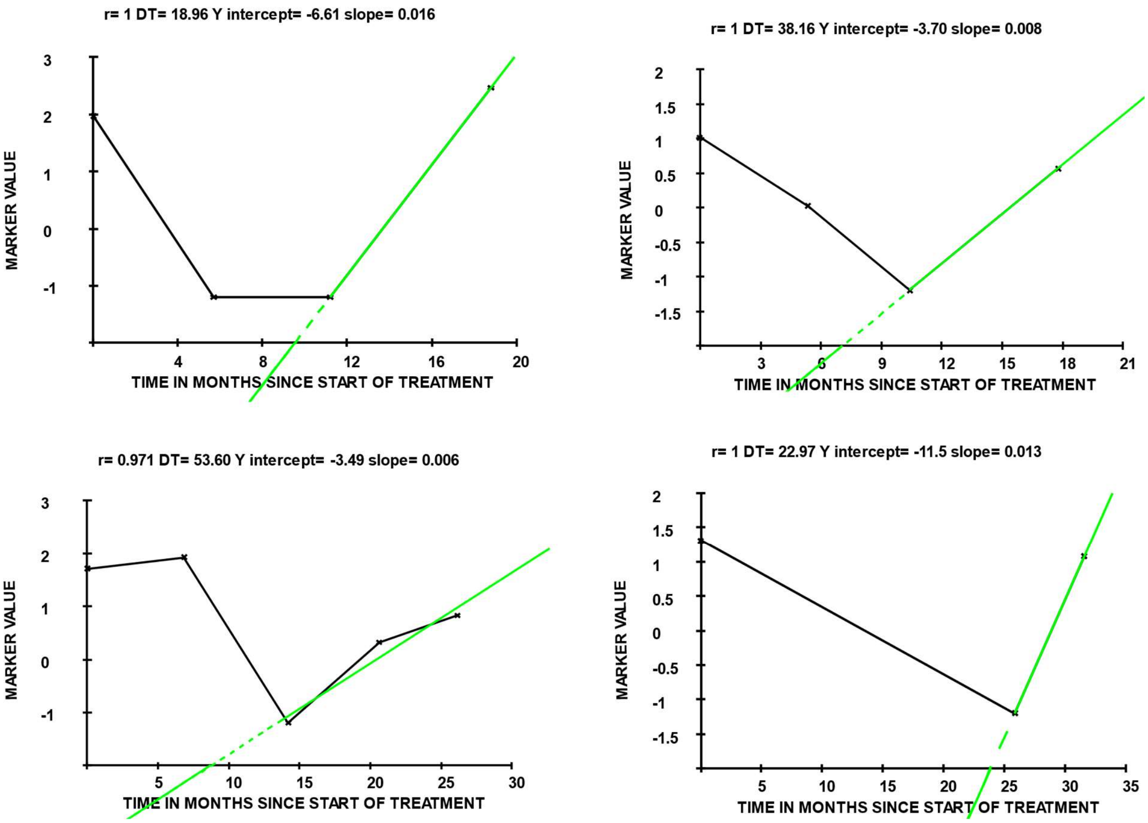

Supplementary figure 14. PFS plotted by the four Limit of Detection categories for MRD negative patients in the MAIA trial.

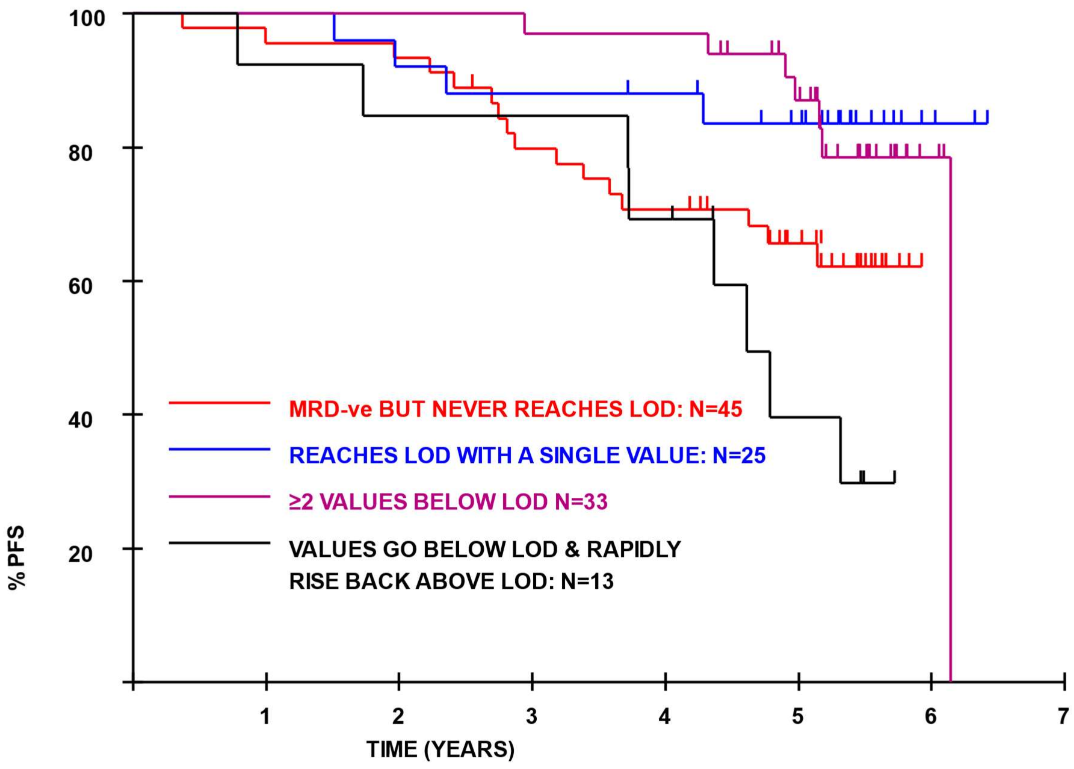

Supplementary figure 15. PFS plotted with updated data by the four Limit of Detection categories for MRD negative patients in the MAIA trial.

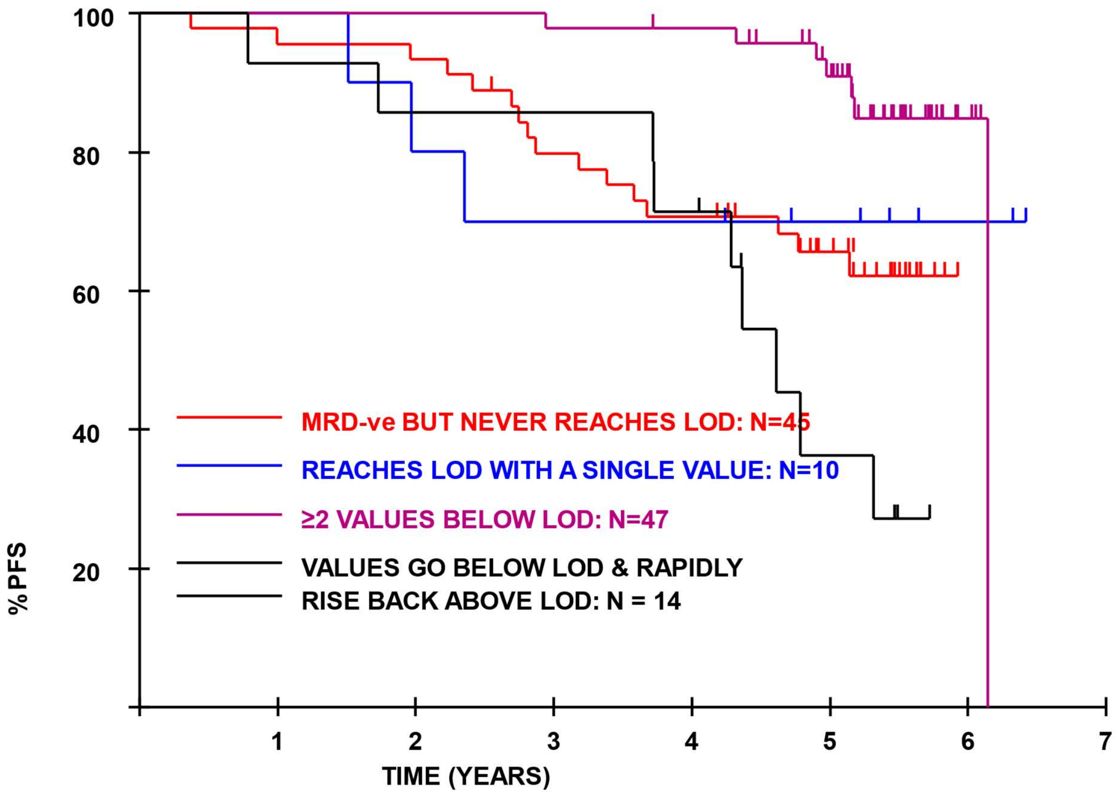

Supplementary Figure 16. Time to achievement of CR or better by arm for those achieving CR or better in the POLLUX trial.

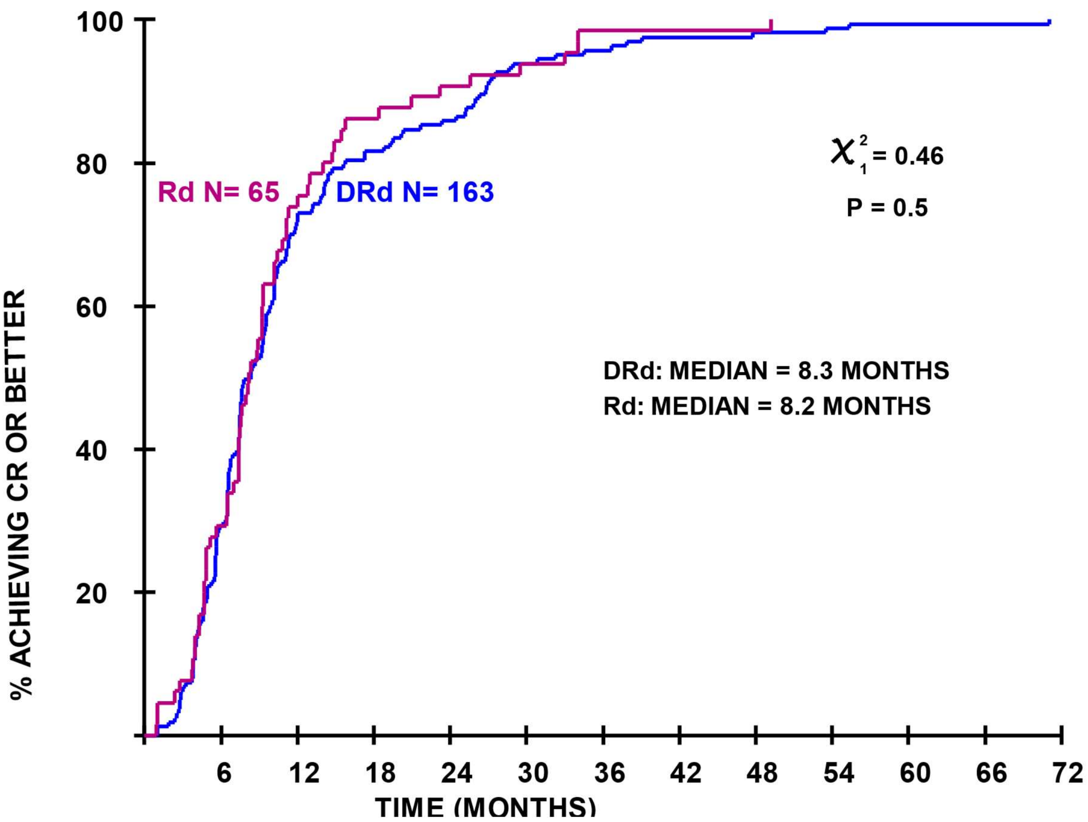

Supplementary Figure 17. Time to achievement of CR or better by arm for those achieving CR or better in the CASTOR trial.

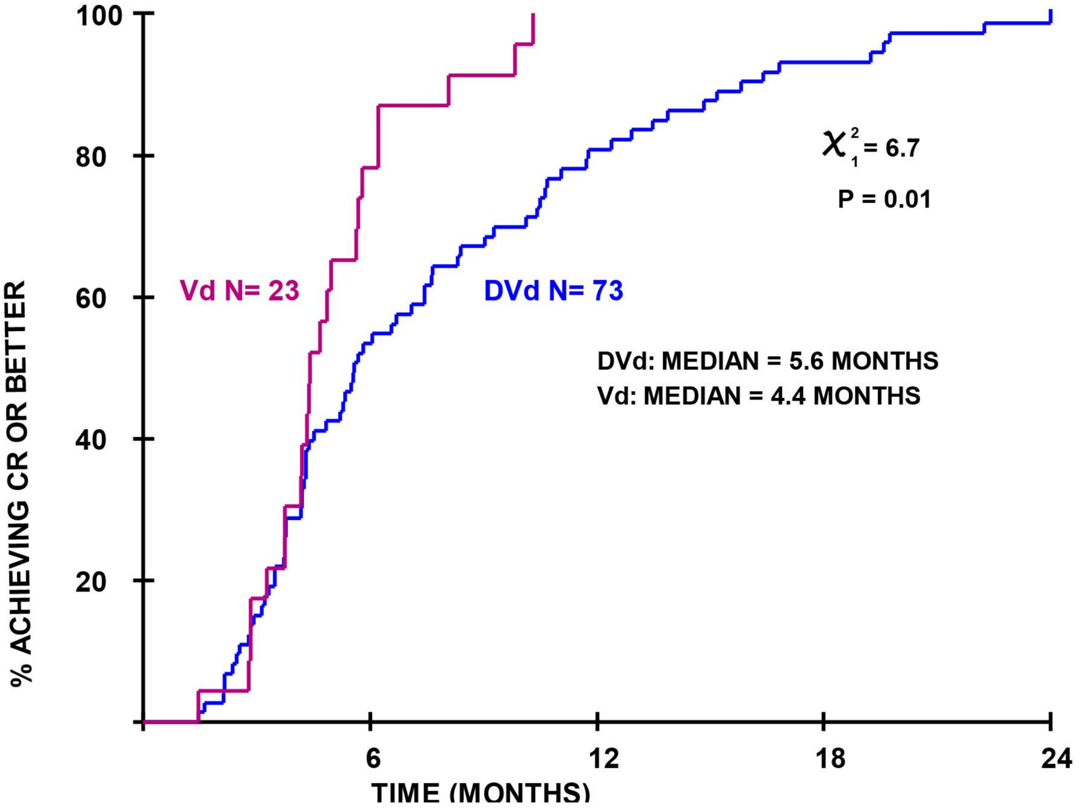

Supplementary figure 18. Model fits to MRD negatives & CRs by arm in the MAIA trial.

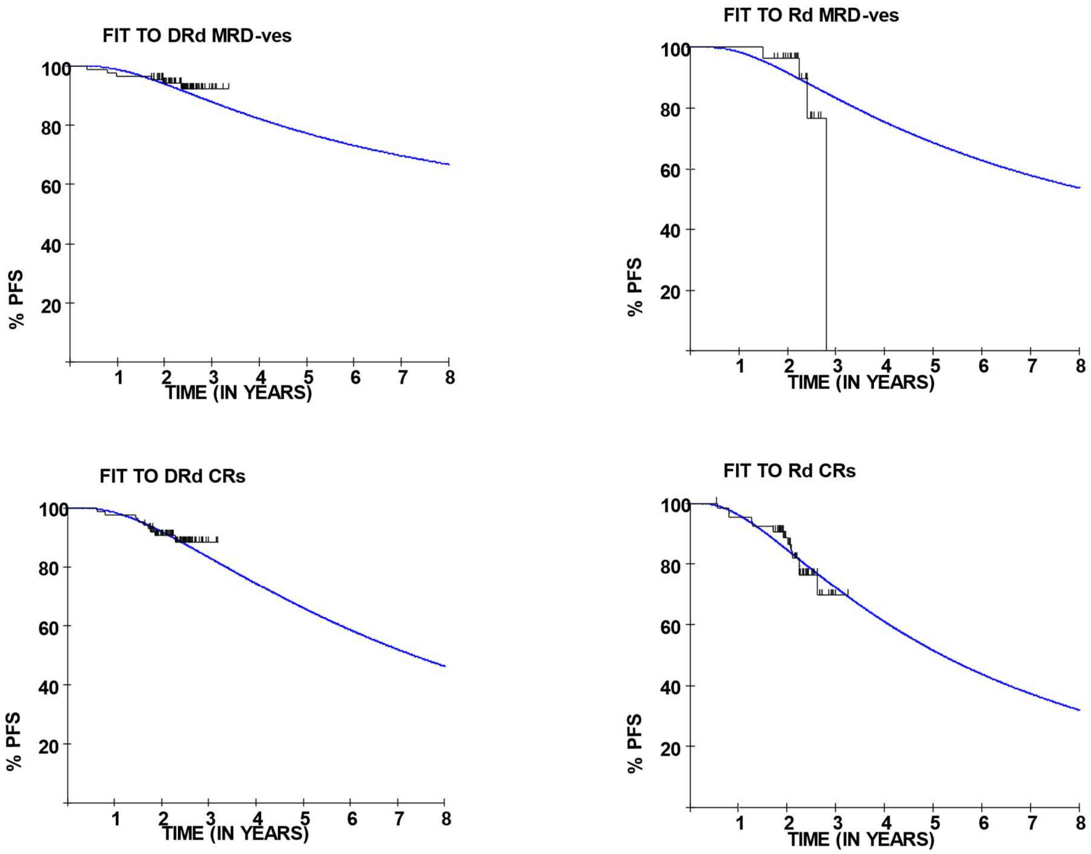

<u>Supplementary figure 19.</u> Model fits to VGPRs & PRs by arm in the MAIA trial.

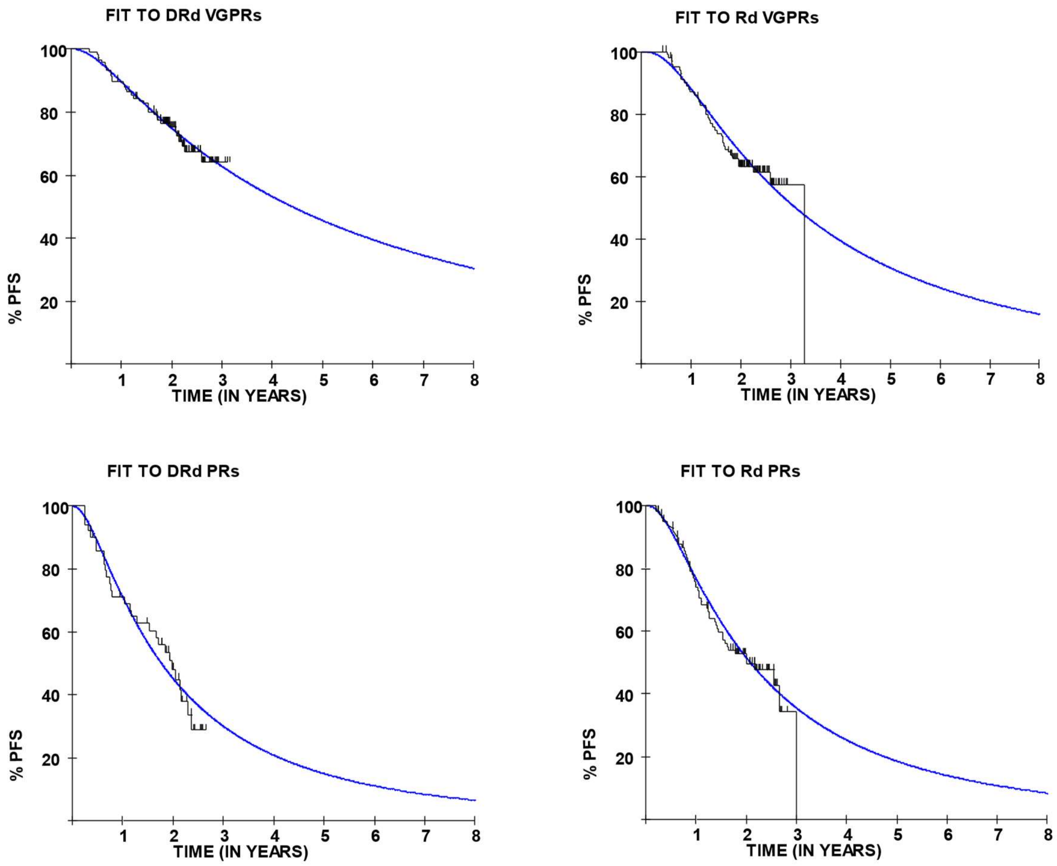

Supplementary figure 20. Model fits to NRs & Unknown responses by arm in the MAIA trial.

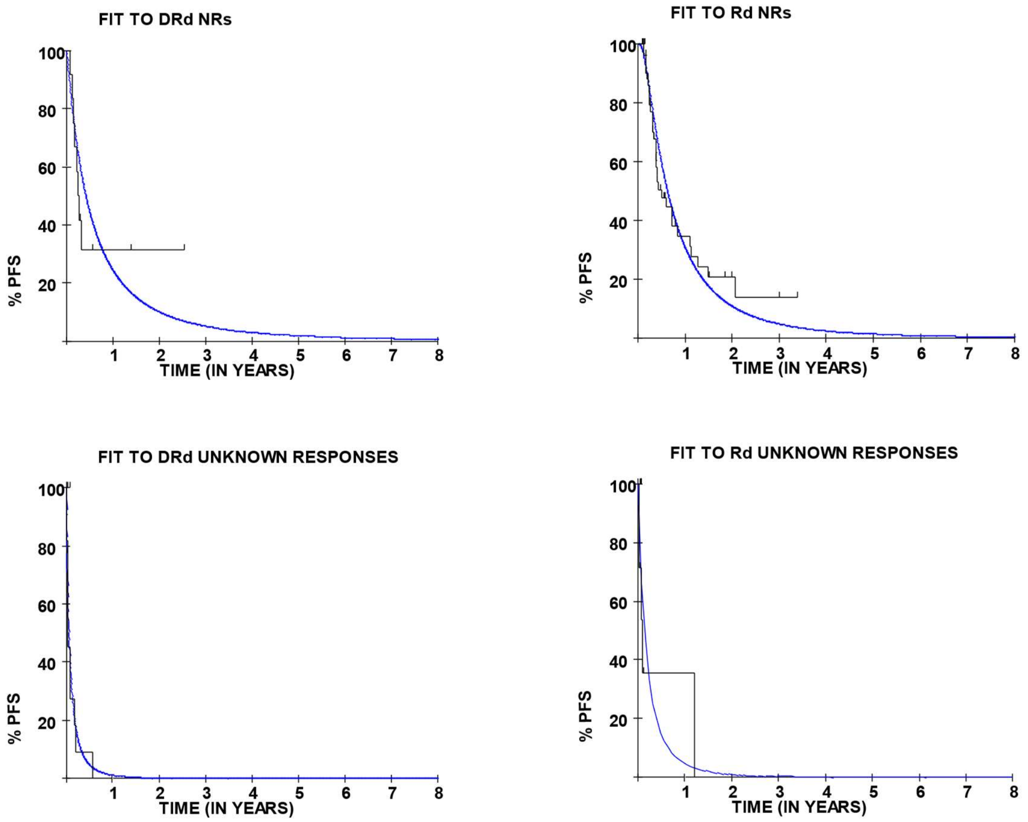

Supplementary figure 21. Overall PFS (95% CI) model fits in both arms with predictions for 3 years beyond currently available data.

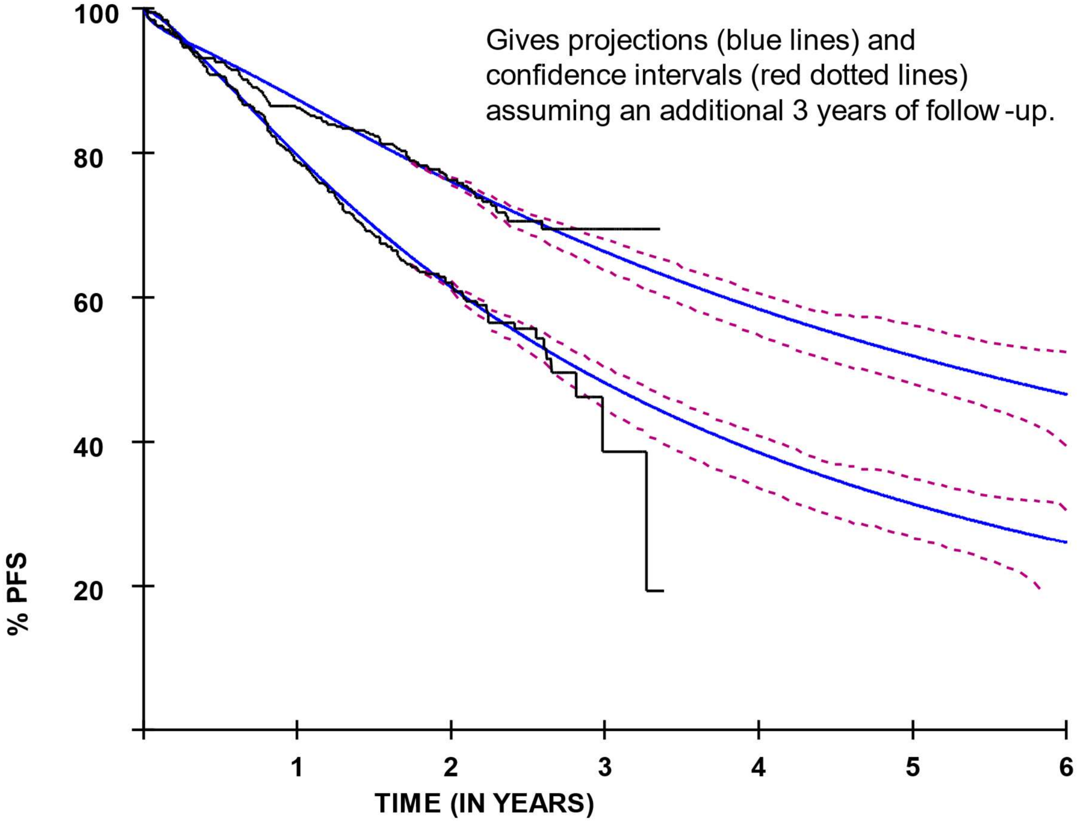

Supplement: Supplementary Data 1 — supplementary online material, tables, figures, mathematics. [file ccr-24-3475_supplementary_data_1_suppds1.pdf]
